# Supplementary material for: Halogen-bonded cocrystallization with phosphorus, arsenic and antimony acceptors
Source: Nat Commun. 2019 Jan 4;10:61. doi: 10.1038/s41467-018-07957-6 (PMC6320372; doi:10.1038/s41467-018-07957-6)
Supplement: Supplementary file 1 — Supplementary Information [file 41467_2018_7957_MOESM1_ESM.pdf]

Supplementary Information

Halogen-bonded cocrystallization with phosphorus, arsenic and antimony acceptors

Lisac et al.

Contents

**1.**	**Supplementary Figures**	2
**2.**	**Supplementary Tables**	21
**3.**	**Supplementary Notes**	25

Supplementary Figures

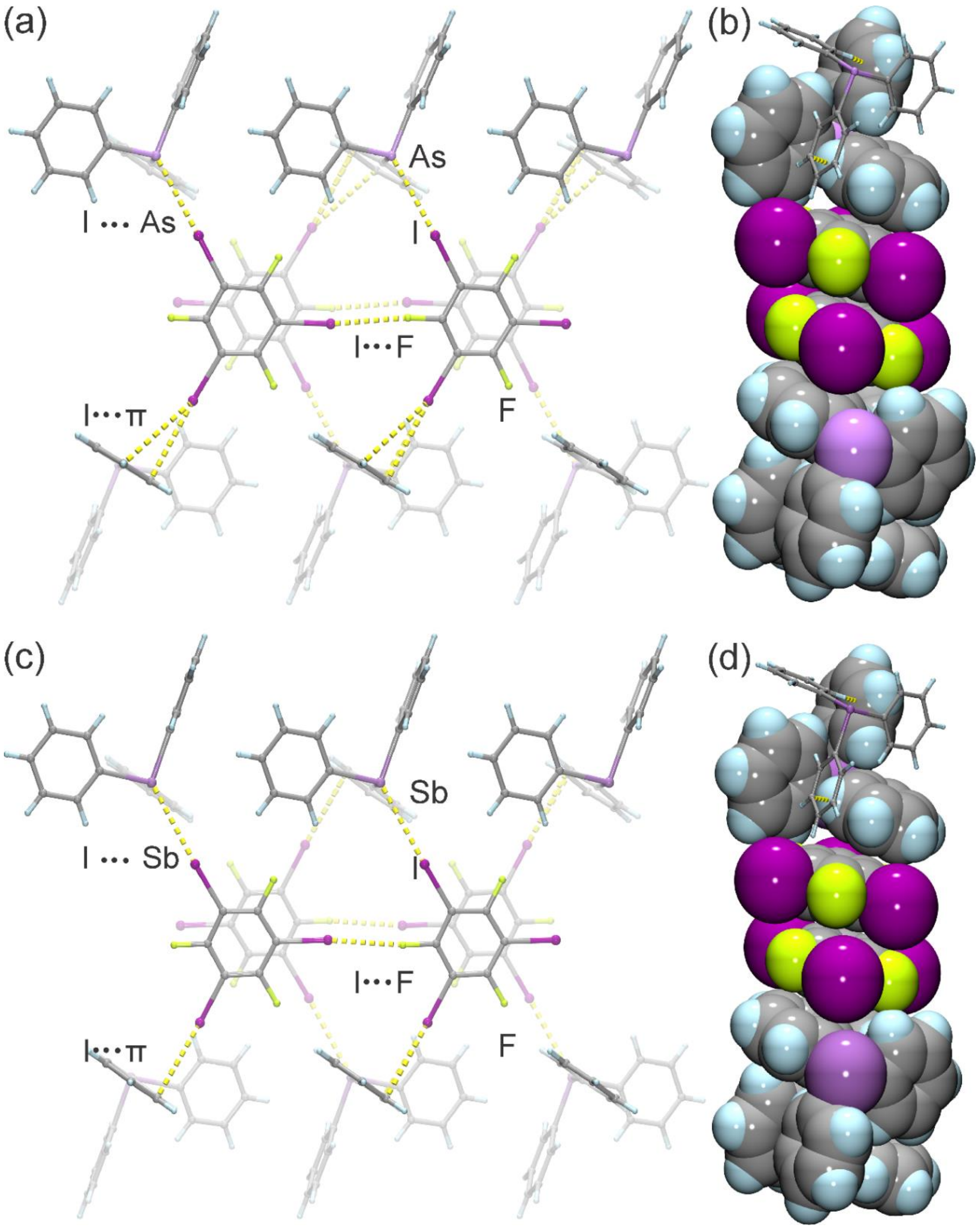

Supplementary Figure 1. Overview of intermolecular contacts and molecular packing in (**tftib**)(**AsPh₃**) and (**tftib**)(**SbPh₃**). (a) In (**tftib**)(**AsPh₃**), each of the iodine atoms of the **tftib** molecule is involved in a short I⋯As interaction with a neighbouring **AsPh₃** unit, an I⋯π contact with another neighbouring **AsPh₃** molecule or a short I⋯F contact to a neighbouring **tftib** molecule. (b) Pairs of **tftib** molecules in the crystal structure of (**tftib**)(**AsPh₃**) stack with phenyl rings of two neighbouring **AsPh₃** moieties; (c) in (**tftib**)(**SbPh₃**) each of the iodine atoms of the **tftib** molecule is involved in a short I⋯Sb interaction with a neighbouring **SbPh₃** unit, an I⋯π contact with another neighbouring **SbPh₃** molecule or a short I⋯F contact to a neighbouring **tftib** molecule. (d) Pairs of **tftib** molecules in the crystal structure of (**tftib**)(**SbPh₃**) stack with phenyl rings of two neighboring **SbPh₃** moieties.

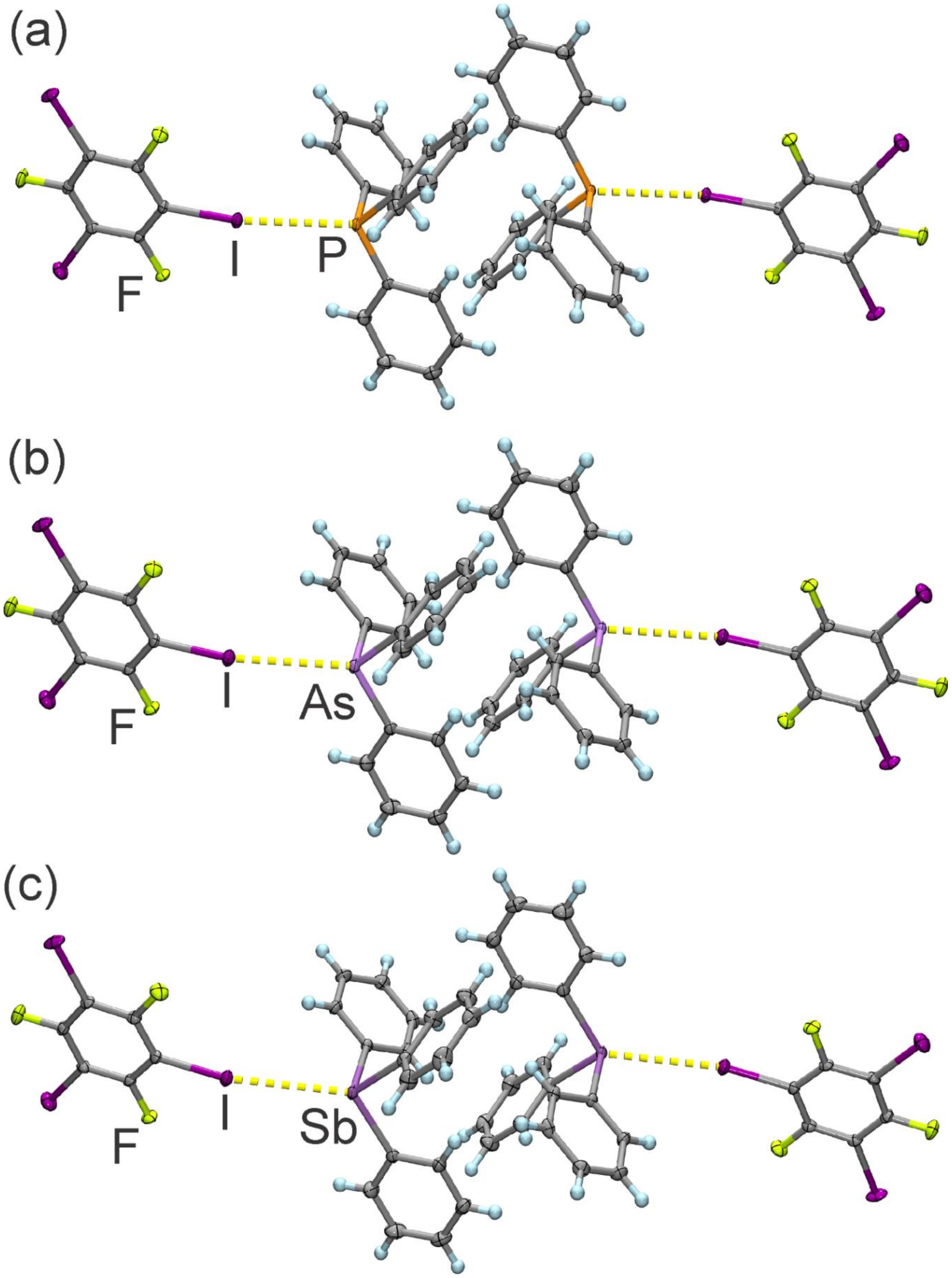

Supplementary Figure 2. Identical views of the phenyl embrace motifs between halogen bond acceptor molecules in cocrystal structures of (a) (**tftib**)(**PPh_3**); (b) (**tftib**)(**$AsPh_3$**) and (c) (**tftib**)(**$SbPh_3$**). Halogen bonds I⋯P, I⋯As and I⋯Sb are also highlighted, as yellow dotted lines.

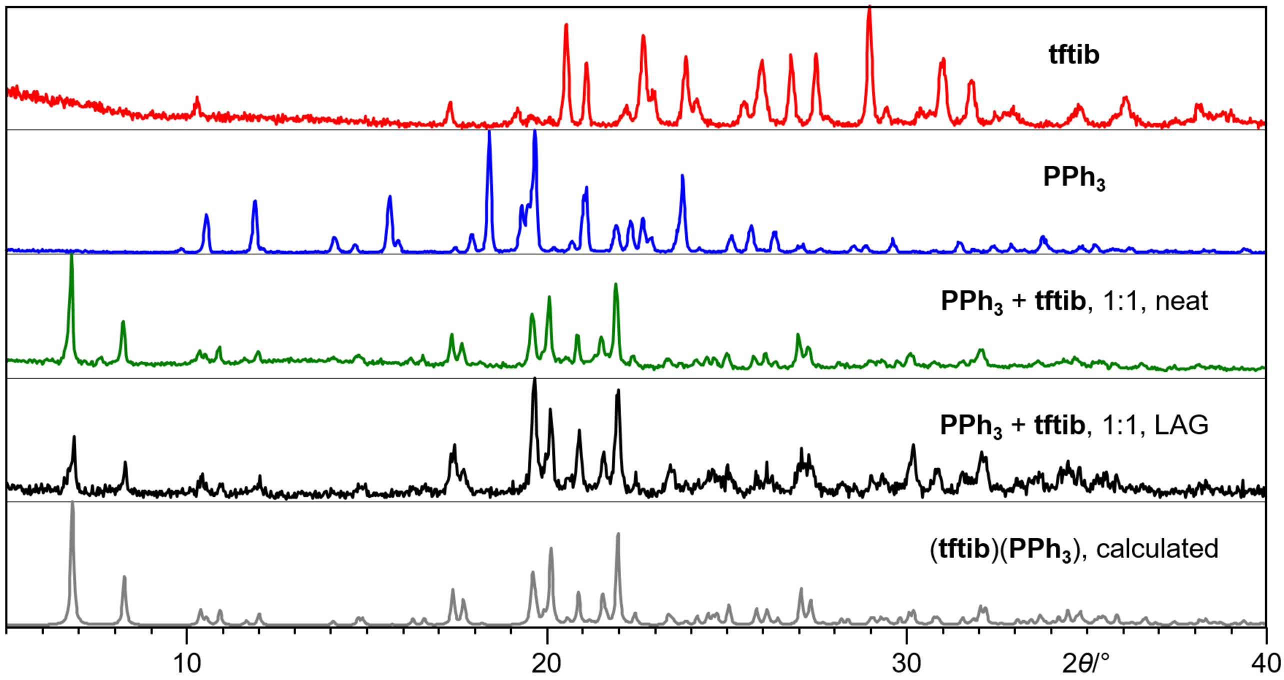

Supplementary Figure 3. (from top to bottom) Powder X-ray diffraction (PXRD) patterns of 1,3,5-trifluoro-2,4,6-triiodobenzene (**tftib**), triphenylphosphine (**PPh_3**), ground mixtures of **PPh_3** and **tftib** – neat and liquid-assisted grinding (LAG), and the pattern simulated from the crystal structure of the cocrystal (**tftib**)(**PPh_3**).

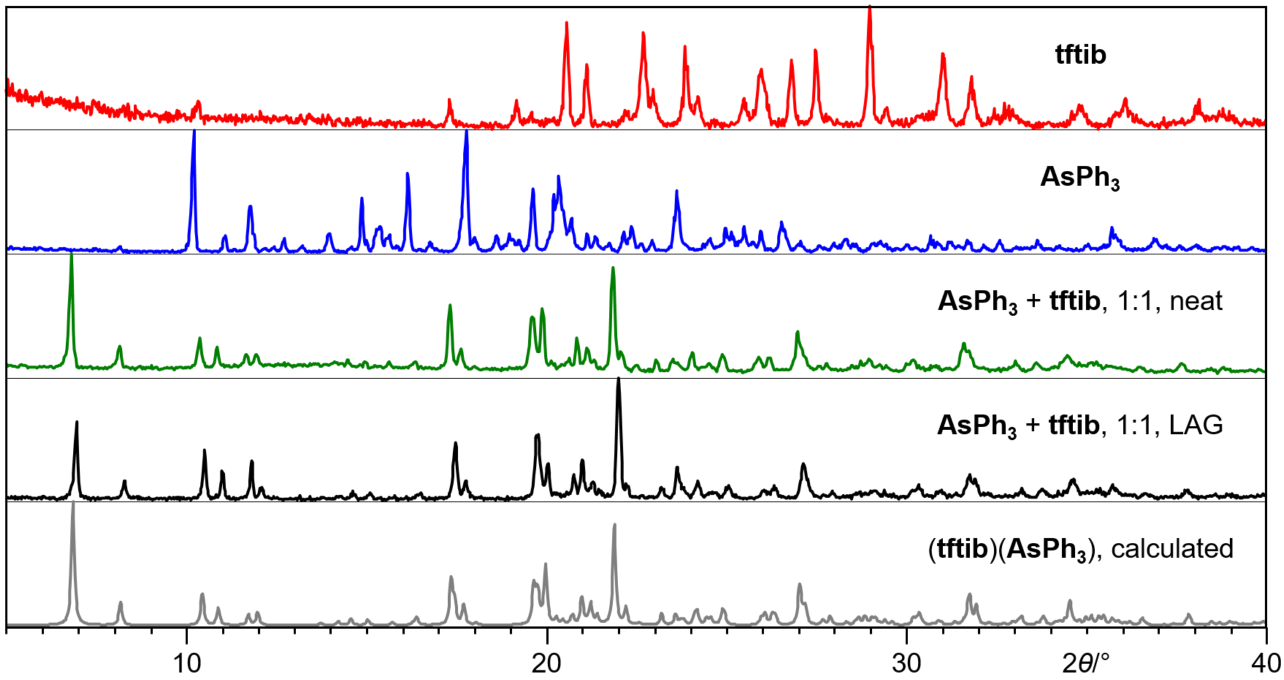

Supplementary Figure 4. (from top to bottom) Powder X-ray diffraction (PXRD) patterns of 1,3,5-trifluoro-2,4,6-triiodobenzene (**tftib**), triphenylarsine (**$AsPh_3$**), ground mixtures of **$AsPh_3$** and **tftib** – neat and liquid-assisted grinding (LAG), and the pattern simulated from the crystal structure of the cocrystal (**tftib**)(**$AsPh_3$**).

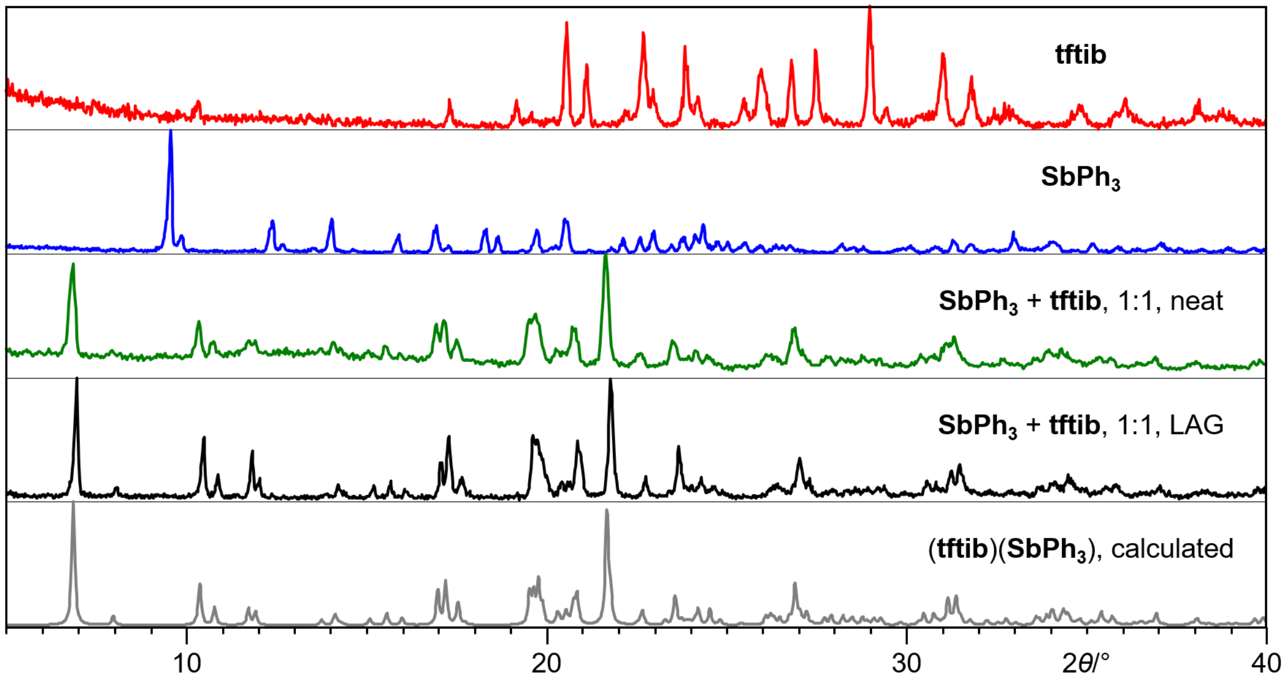

Supplementary Figure 5. (from top to bottom) Powder X-ray diffraction (PXRD) patterns of 1,3,5-trifluoro-2,4,6-triiodobenzene (**tftib**), triphenylstibine (**$SbPh_3$**), ground mixtures of **$SbPh_3$** and **tftib** – neat and liquid-assisted grinding (LAG), and the pattern simulated from the crystal structure of the cocrystal (**tftib**)(**$SbPh_3$**).

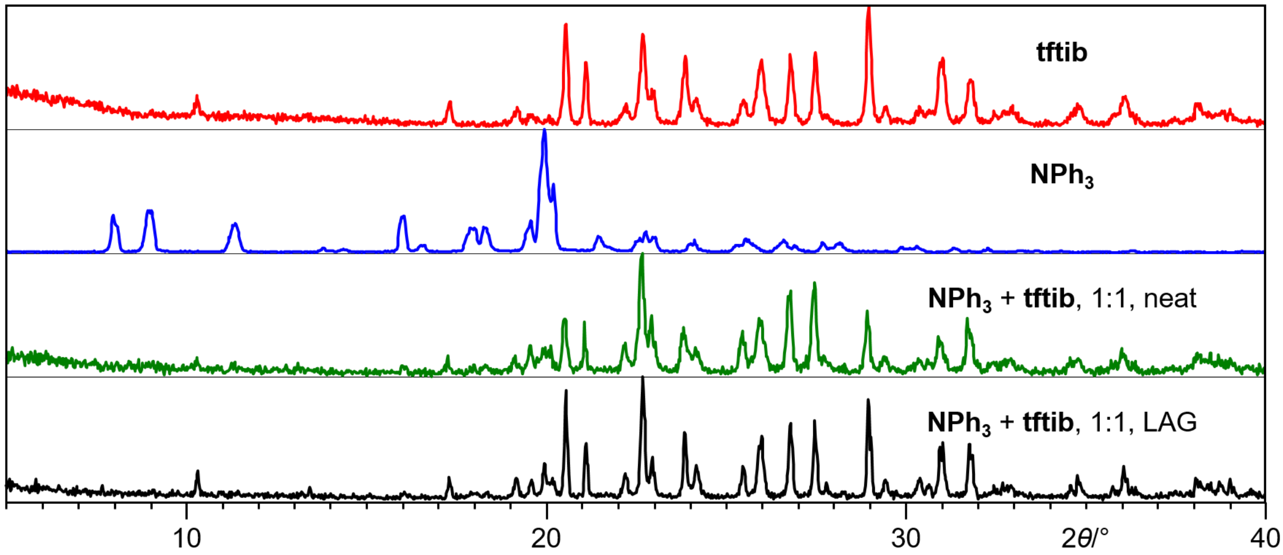

Supplementary Figure 6. (from top to bottom) Powder X-ray diffraction (PXRD) patterns of 1,3,5-trifluoro-2,4,6-triiodobenzene (**tftib**), triphenylamine (**NPh_3**) and the ground mixtures of **NPh_3** and **tftib** – neat and liquid-assisted grinding (LAG).

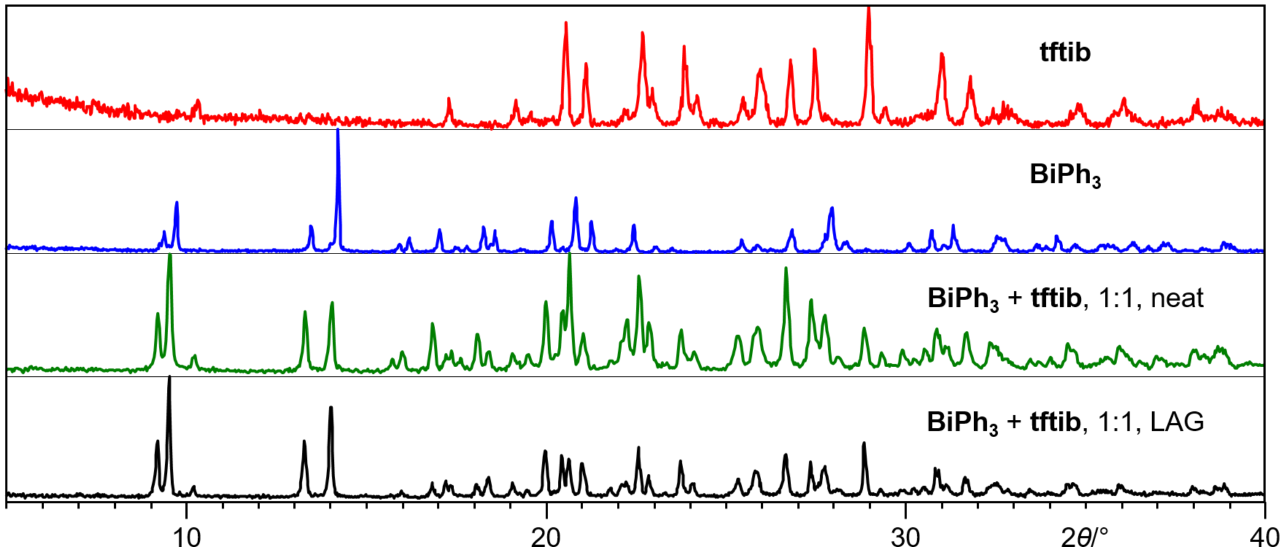

Supplementary Figure 7. (from top to bottom) Powder X-ray diffraction (PXRD) patterns of 1,3,5-trifluoro-2,4,6-triiodobenzene (**tftib**), triphenylbismuth (**$BiPh_3$**) and the ground mixtures of **$BiPh_3$** and **tftib** – neat and liquid-assisted grinding (LAG).

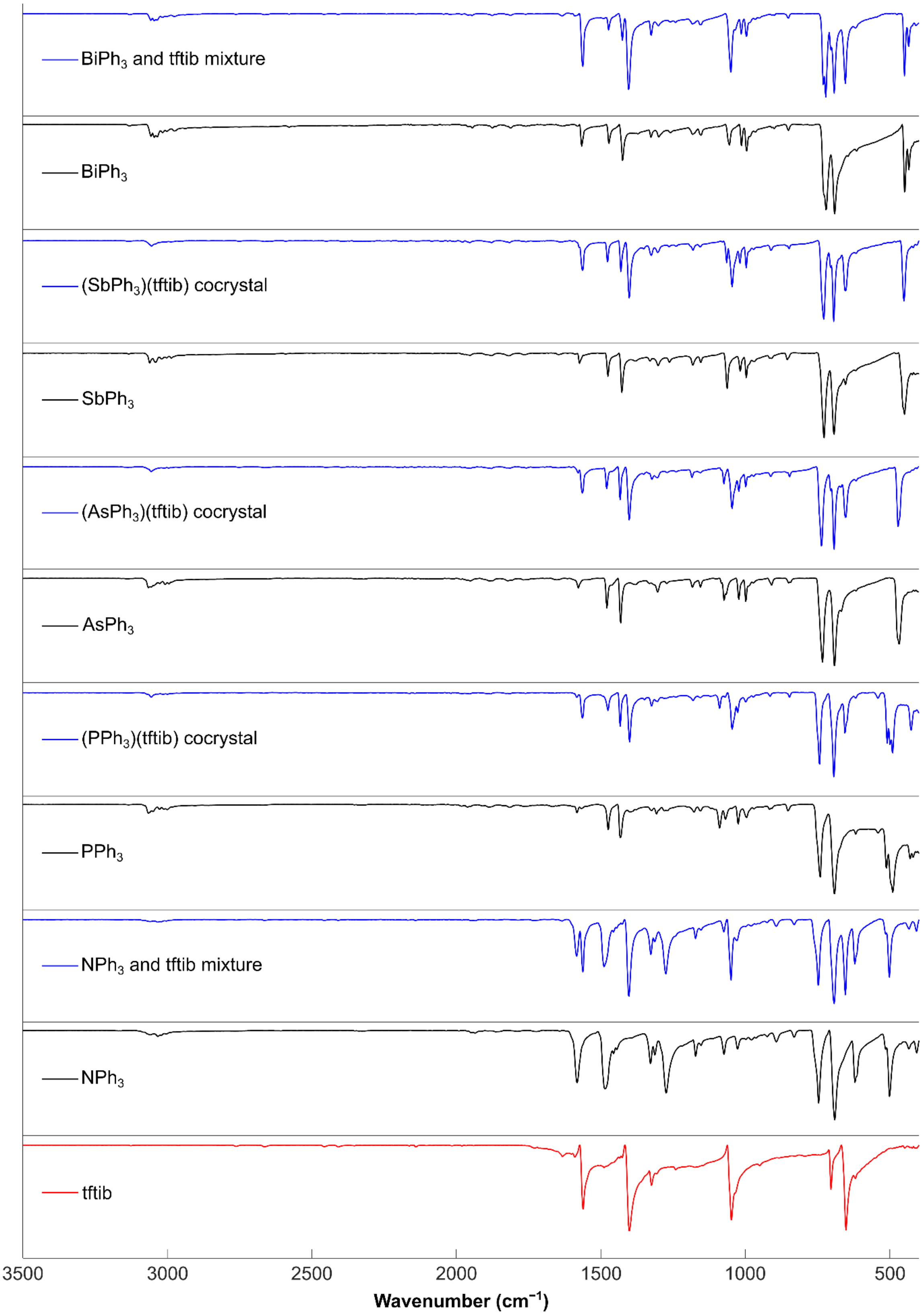

Supplementary Figure 8. (from top to bottom) ATR FT-IR spectra of the mixture of **BiPh$_3$** and **tftib**, pure **BiPh$_3$**, cocrystal (**SbPh$_3$**)(**tftib**), pure **SbPh$_3$**, cocrystal (**AsPh$_3$**)(**tftib**), pure **AsPh$_3$**, cocrystal (**PPh$_3$**)(**tftib**), pure **PPh$_3$**, mixture of **NPh$_3$** and **tftib**, pure **NPh$_3$** and pure **tftib**.

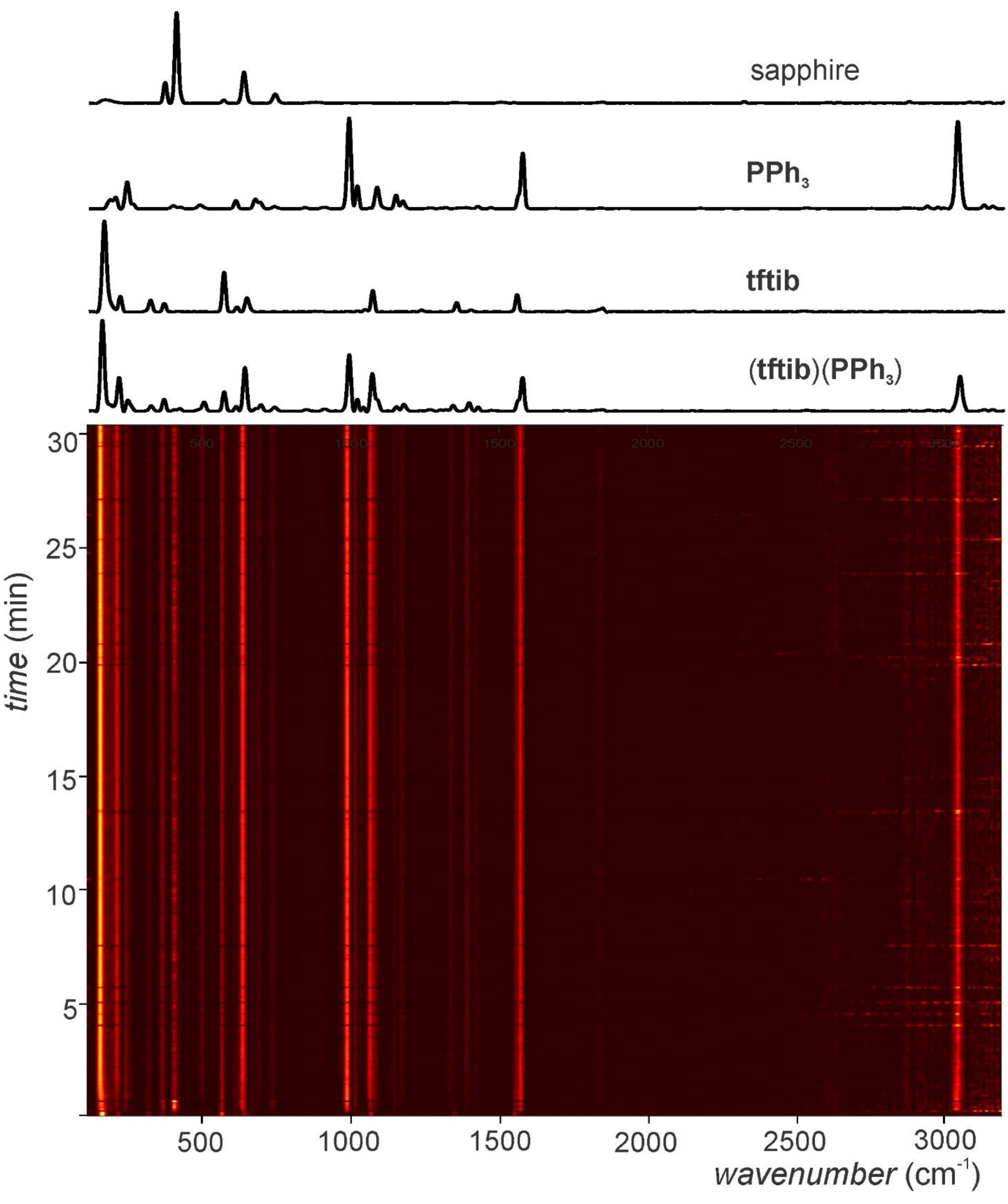

Supplementary Figure 9. Time-resolved Raman spectrum for the mechanochemical neat milling reaction of **PPh_3** and **tftib**. The normalized spectra for reaction components, sapphire milling vessel and the cocrystal product are provided for reference.

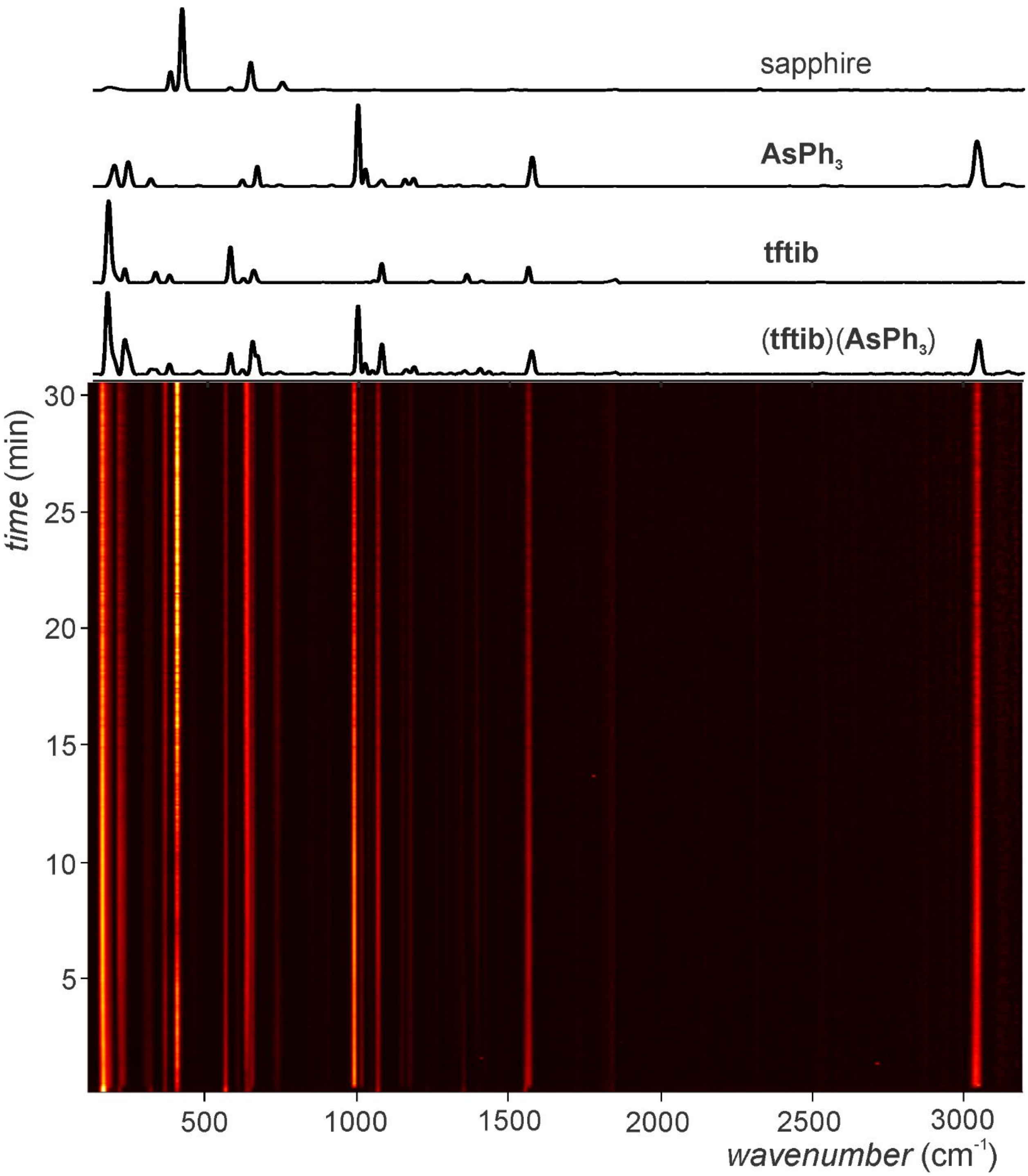

Supplementary Figure 10. Time-resolved Raman spectrum for the mechanochemical neat milling reaction of **$AsPh_3$** and **tftib**. The normalized spectra for reaction components, sapphire milling vessel and the cocrystal product are provided for reference.

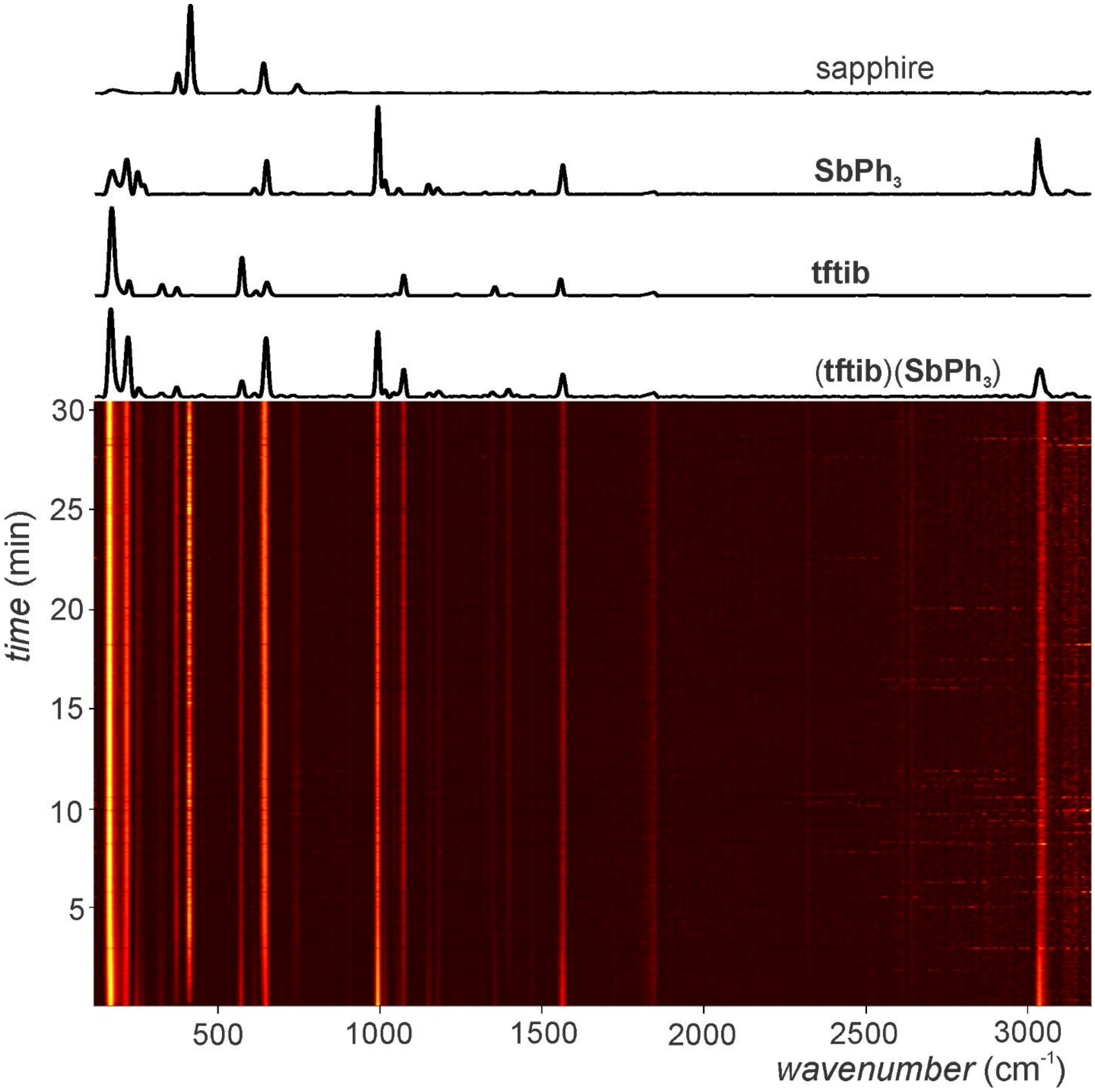

Supplementary Figure 11. Time-resolved Raman spectrum for the mechanochemical neat milling reaction of **$SbPh_3$** and **tftib**. The normalized spectra for reaction components, sapphire milling vessel and the cocrystal product are provided for reference.

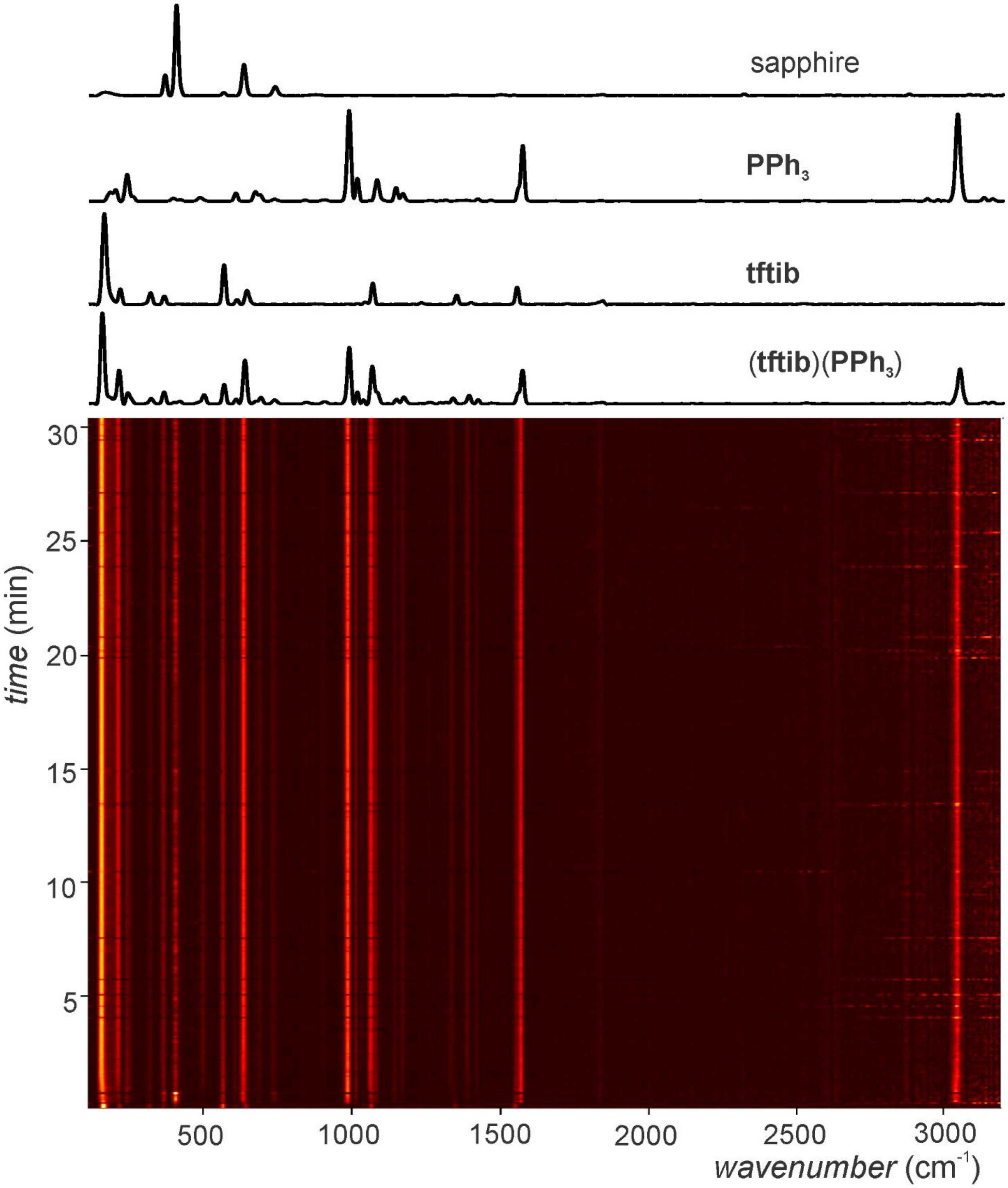

Supplementary Figure 12. Time-resolved Raman spectrum for the mechanochemical LAG reaction of **PPh_3** and **tftib**. The normalized spectra for reaction components, sapphire milling vessel and the cocrystal product are provided for reference.

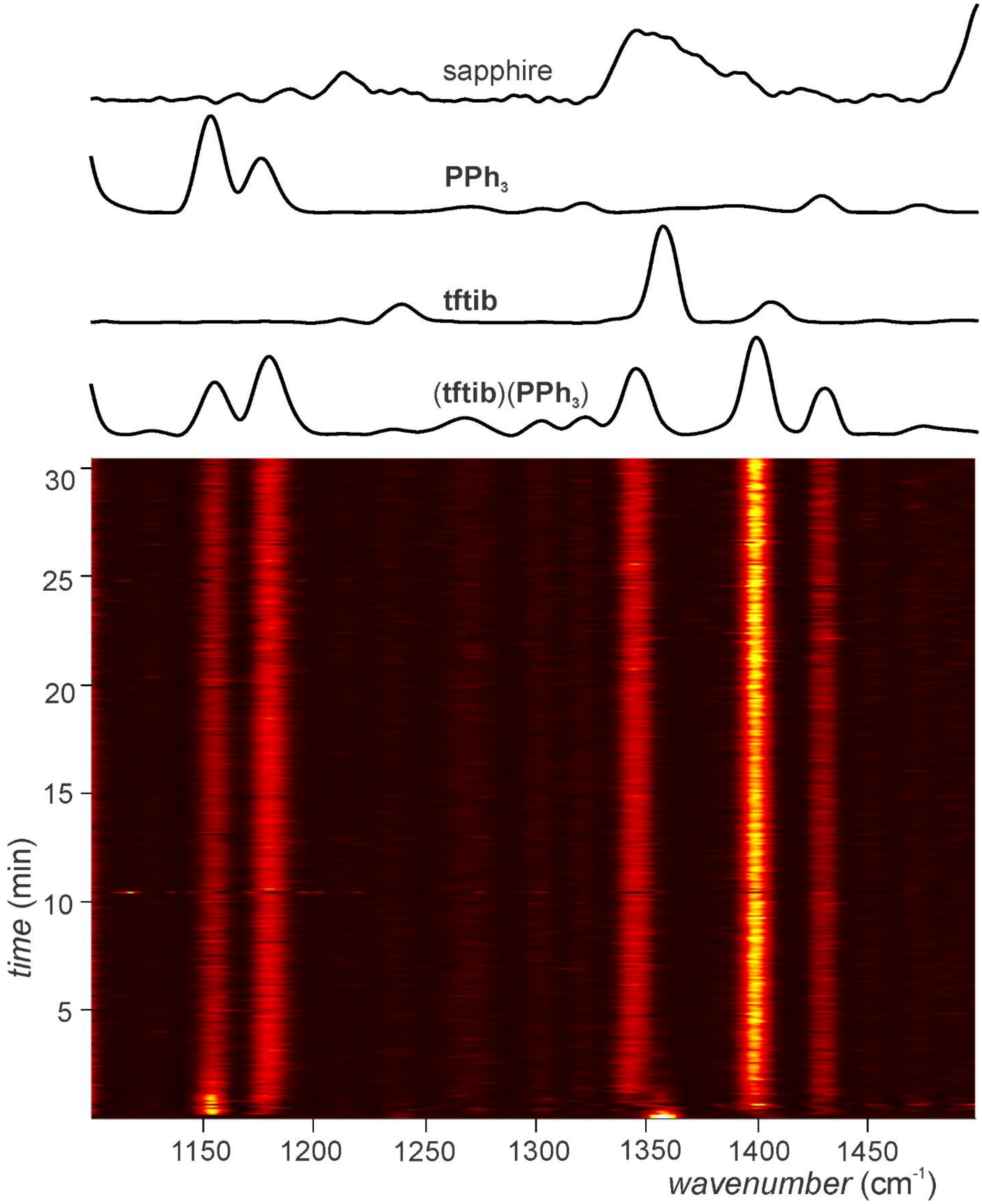

Supplementary Figure 13. Segment of the time-resolved Raman spectrum for the mechanochemical LAG reaction of **PPh_3** and **tftib**, highlighting the spectral area between 1100 cm^{-1} and 1500 cm^{-1}. The normalized spectra for reaction components, sapphire milling vessel and the cocrystal product are provided for reference.

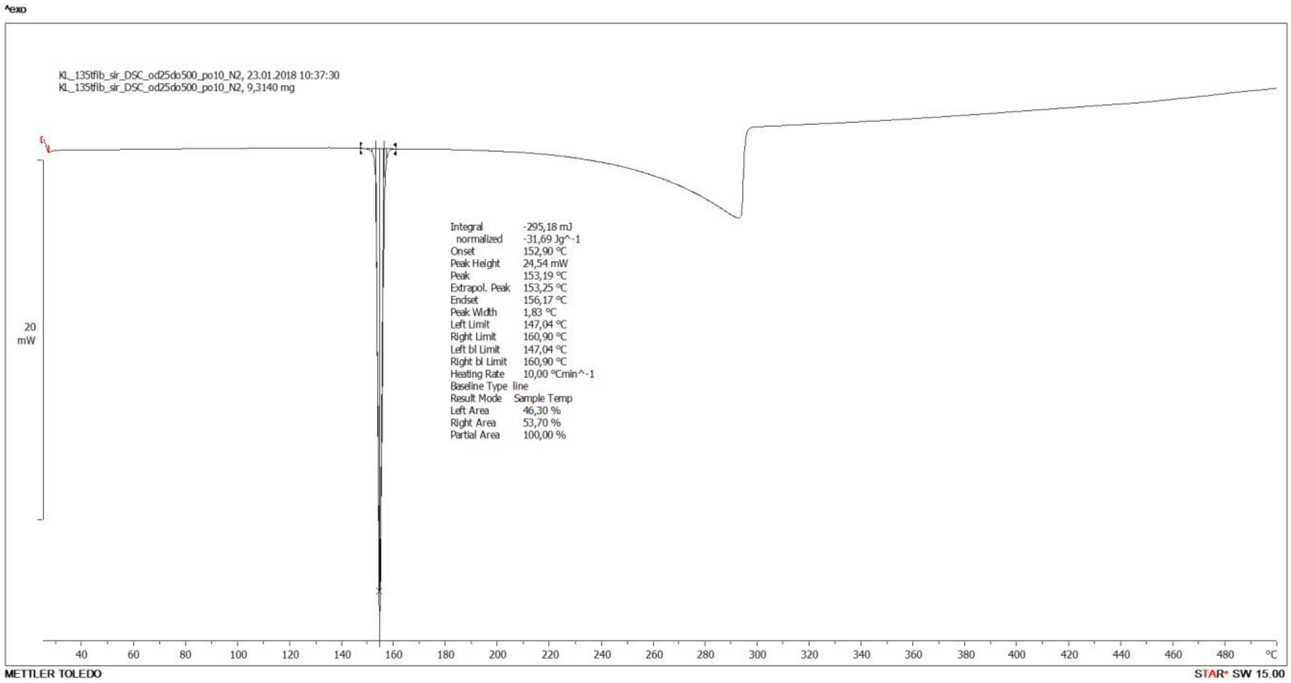

Supplementary Figure 14. DSC thermogram of **tftib** (25–500 °C, heating rate 10 °C min^{-1}).

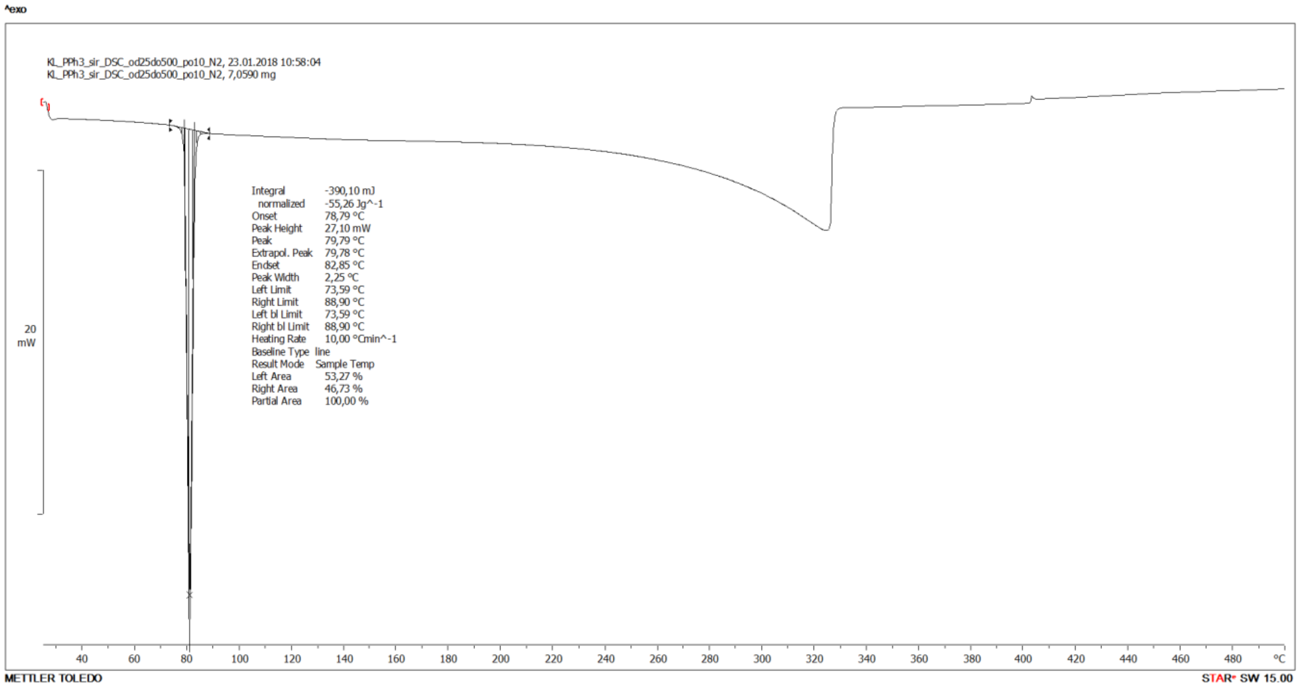

Supplementary Figure 15. DSC thermogram of **PPh_3** (25–500 °C, heating rate 10 °C min^{-1}).

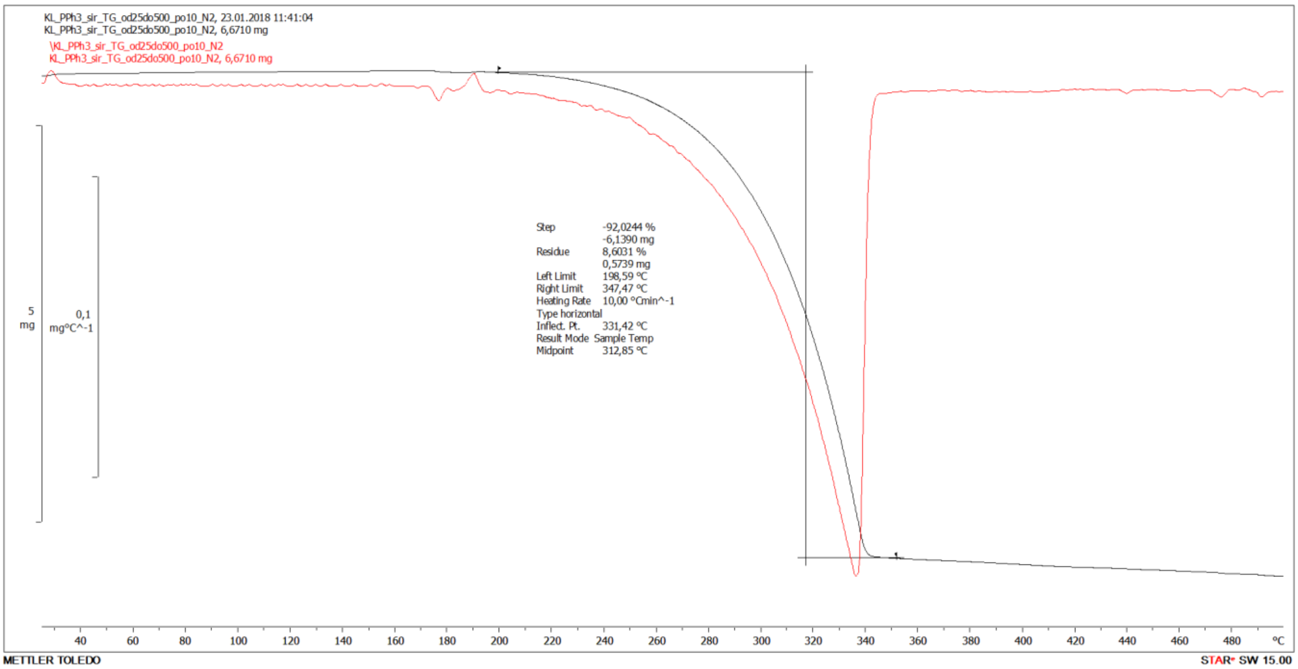

Supplementary Figure 16. TGA thermogram of **PPh3** (25–500 °C, heating rate 10 °C min^{-1}).

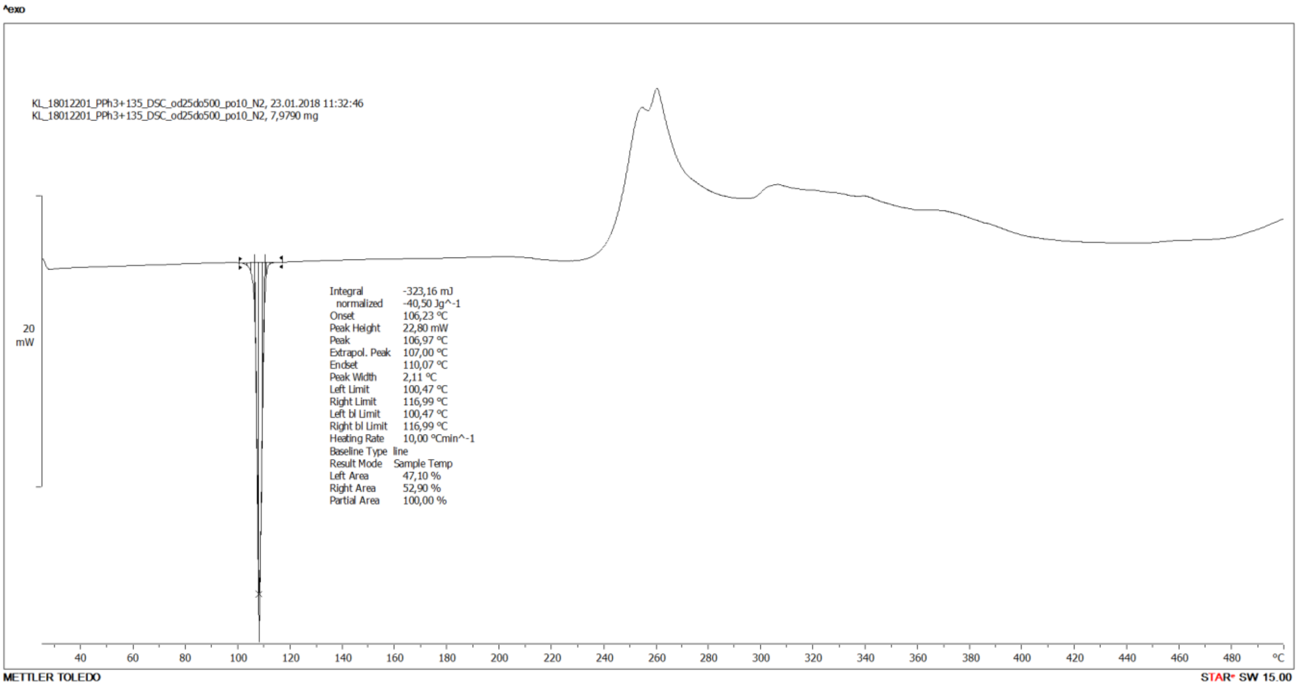

Supplementary Figure 17. DSC thermogram of (**tftib**)(**PPh3**) (25–500 °C, heating rate 10 °C min^{-1}).

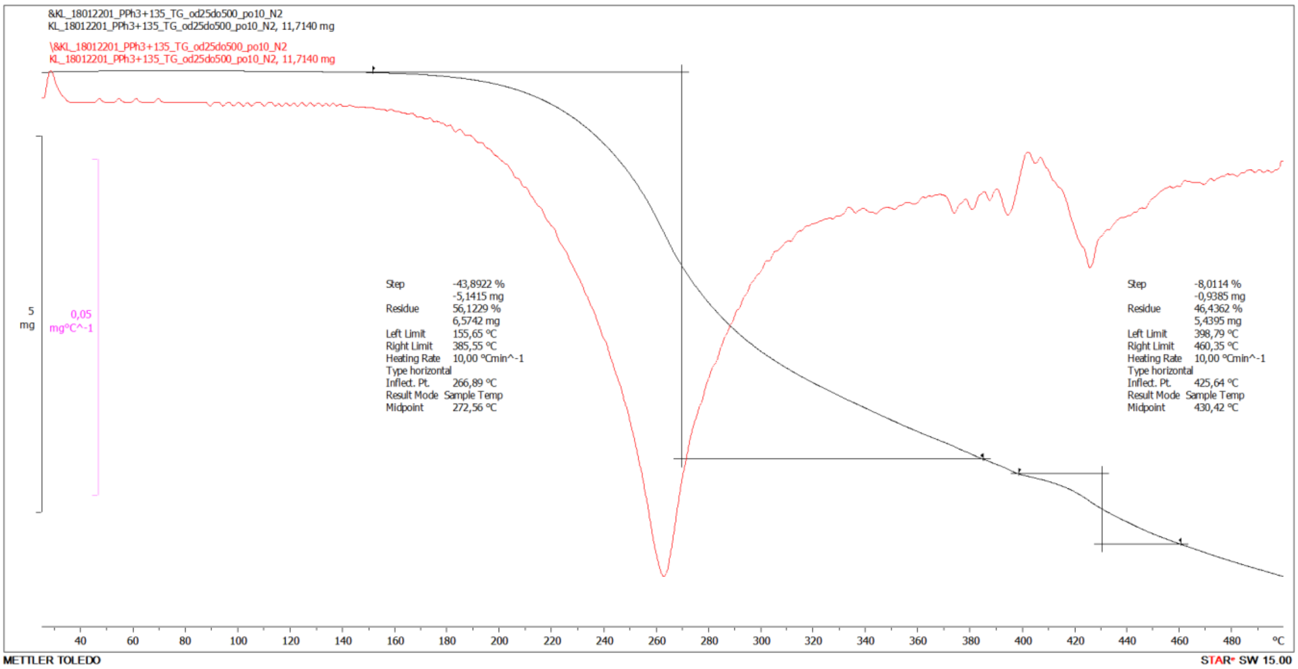

Supplementary Figure 18. TGA thermogram of (**tftib**)(**PPh3**) (25–500 °C, heating rate 10°C min^{-1}).

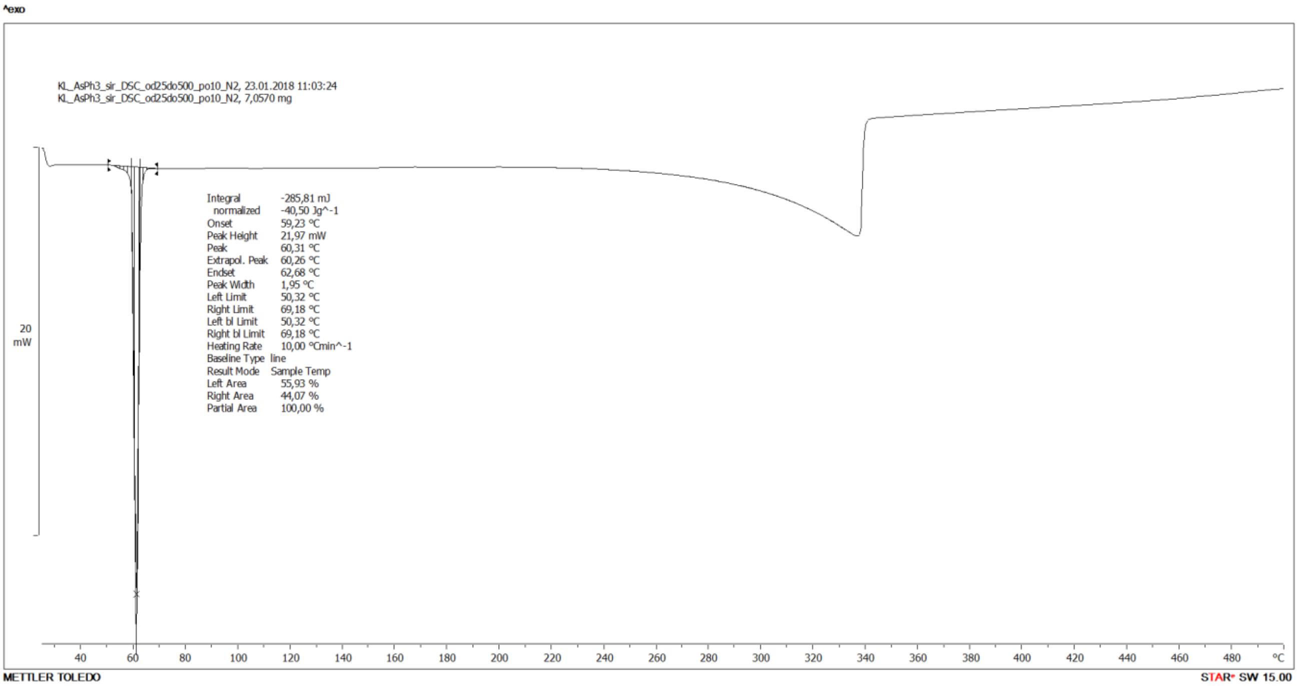

Supplementary Figure 19. DSC thermogram of **AsPh3** (25–500 °C, heating rate 10°C min^{-1}).

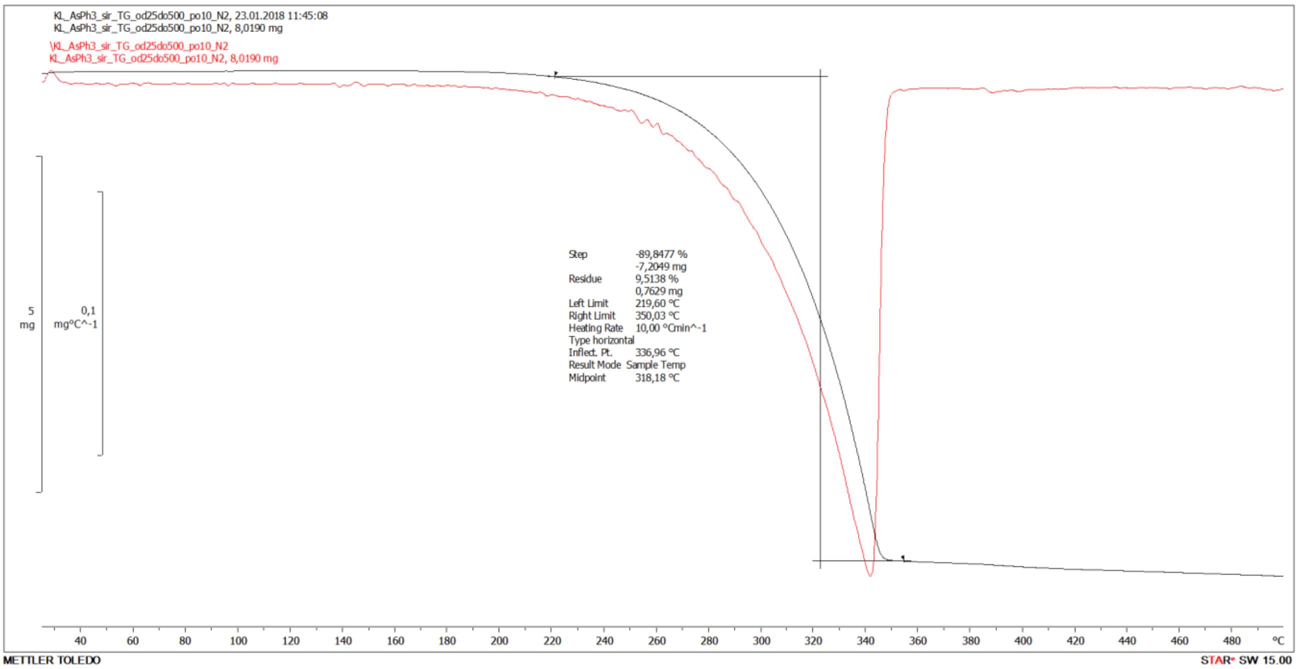

Supplementary Figure 20. TGA thermogram of **$AsPh_3$** (25–500 °C, heating rate 10°C min^{-1}).

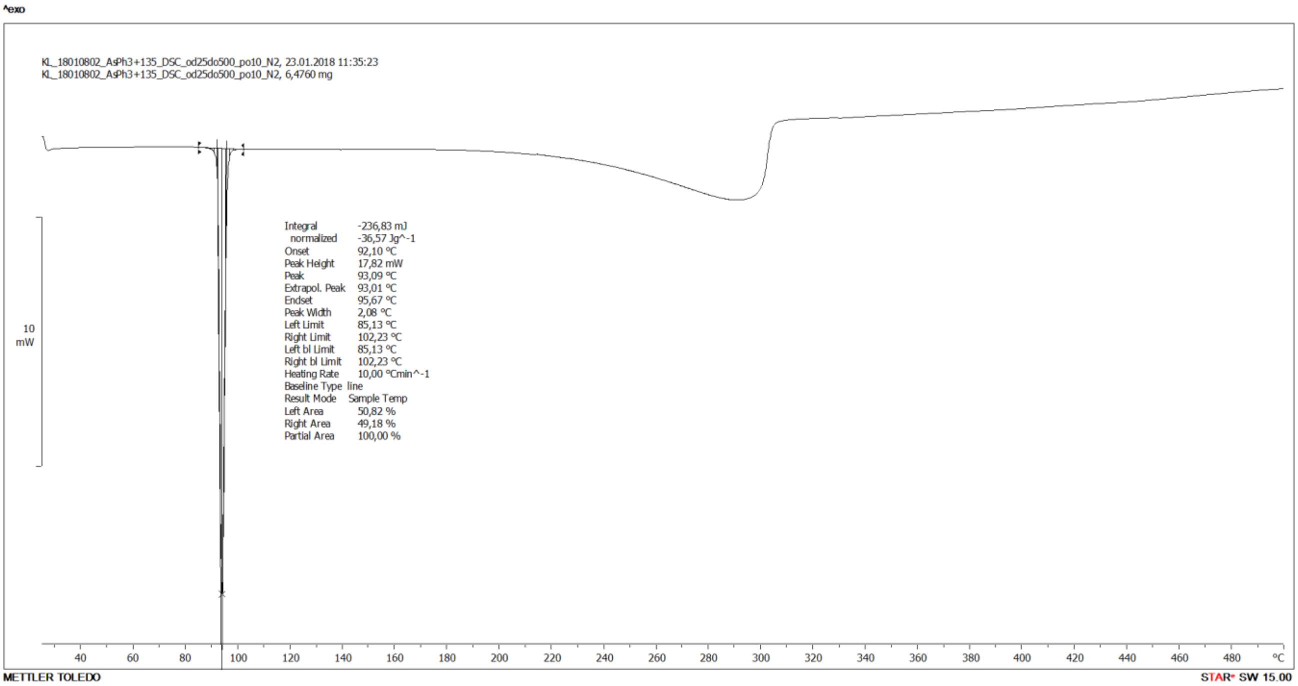

Supplementary Figure 21. DSC thermogram of (**tftib**)(**$AsPh_3$**) (25–500 °C, heating rate 10°C min^{-1}).

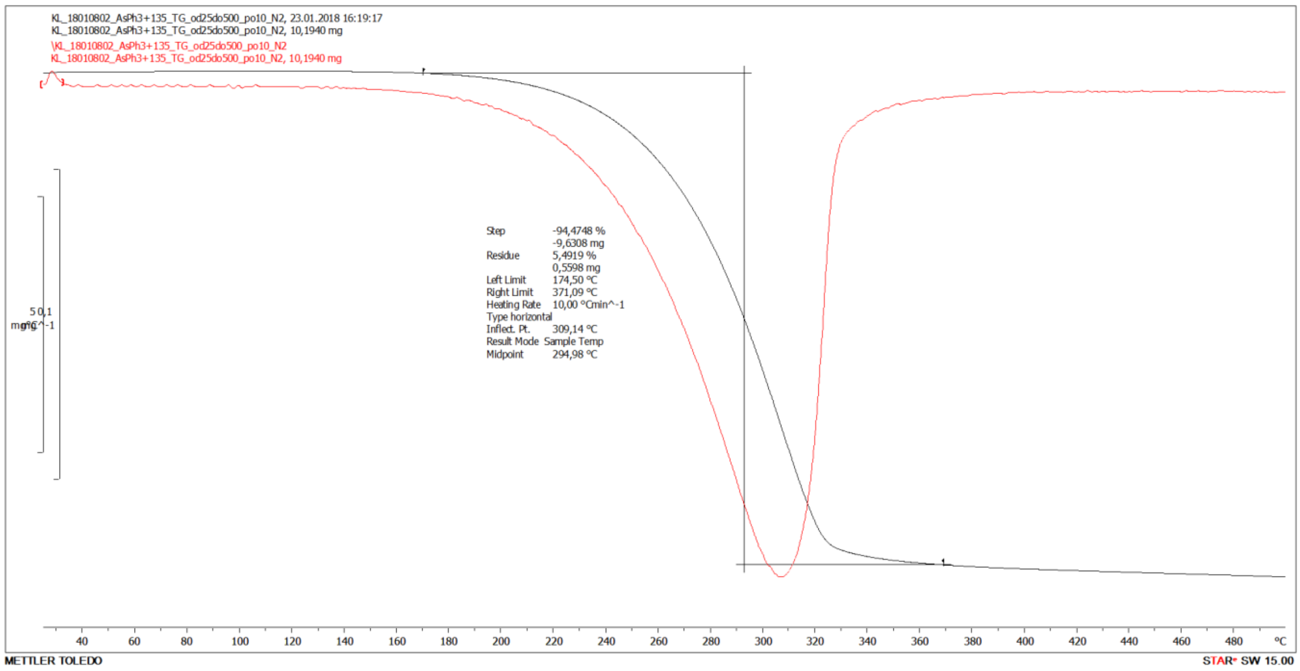

Supplementary Figure 22. TGA thermogram of (**tftib**)(**AsPh$_3$**) (25–500 °C, heating rate 10°C min^{-1}).

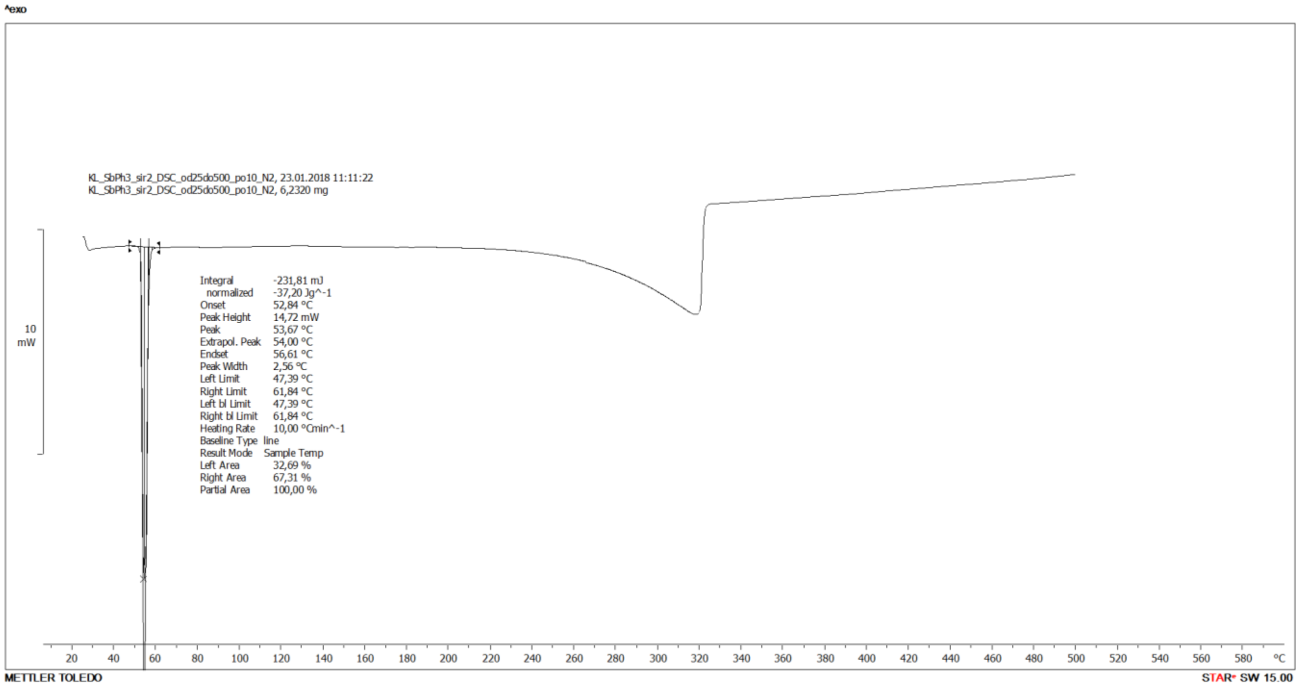

Supplementary Figure 23. DSC thermogram of **SbPh$_3$** (25–500 °C, heating rate 10 °C min^{-1}).

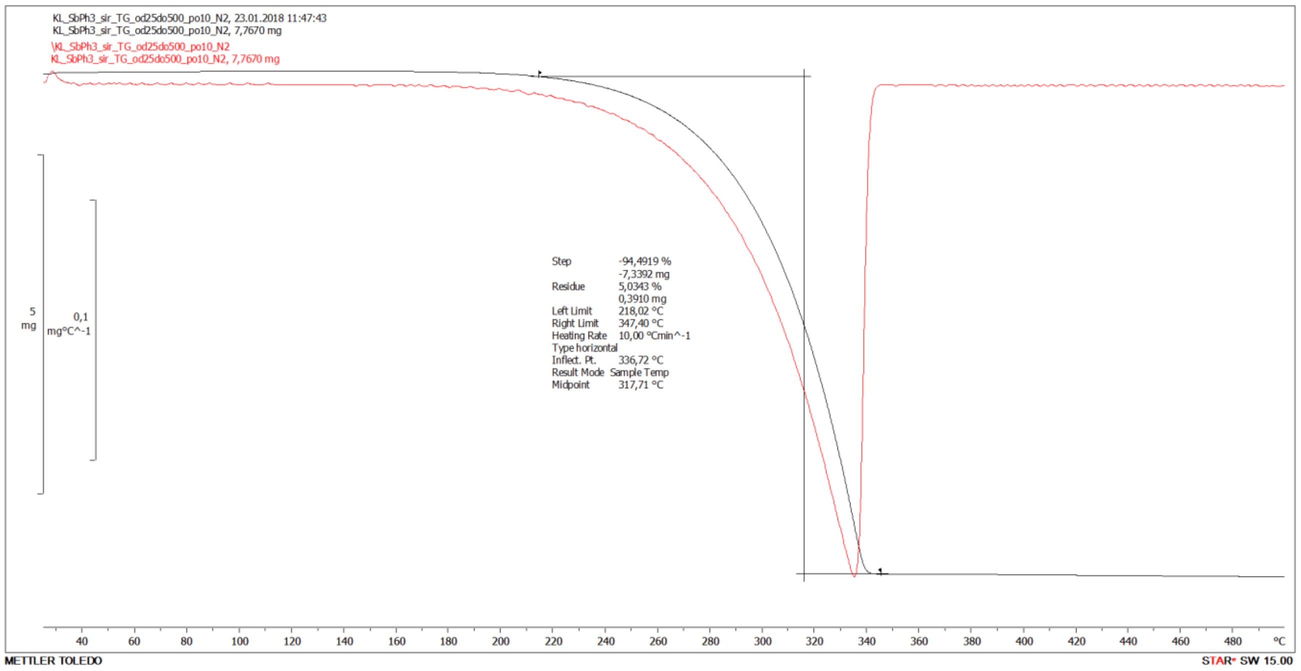

Supplementary Figure 24. TGA thermogram of **$SbPh_3$** (25–500 °C, heating rate 10°C min^{-1}).

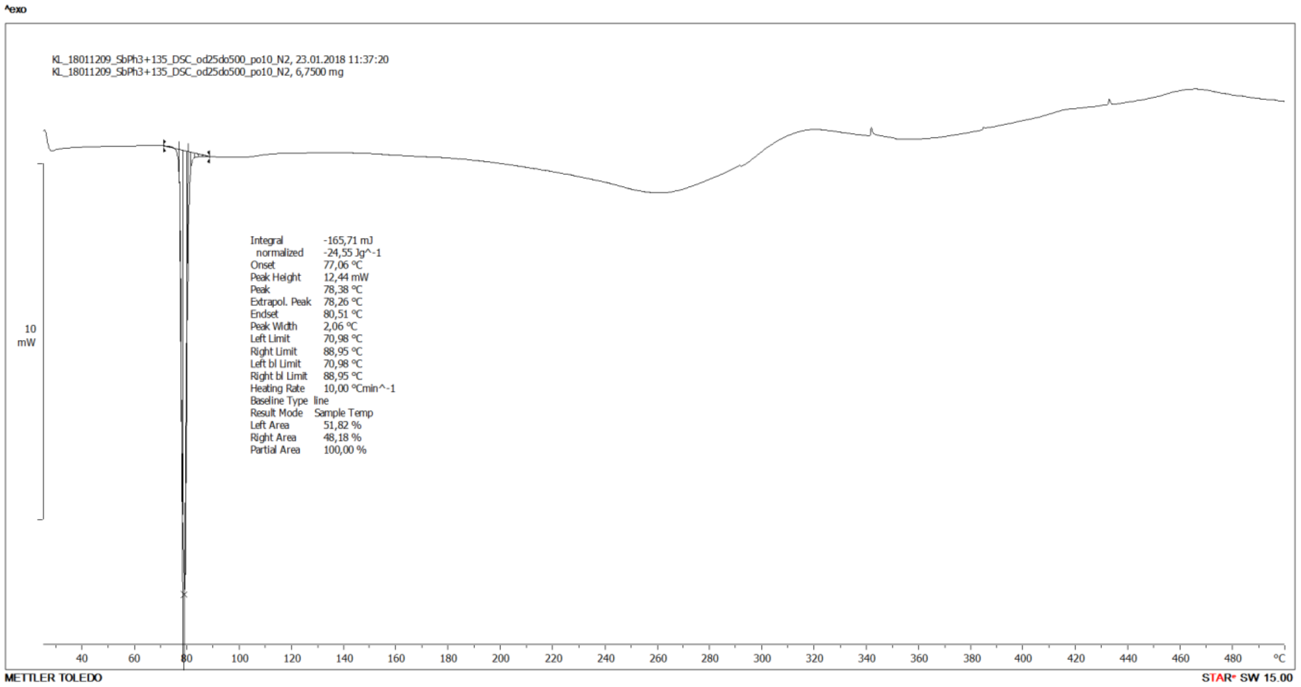

Supplementary Figure 25. DSC thermogram of (**tftib**)(**$SbPh_3$**) (25–500 °C, heating rate 10°C min^{-1}).

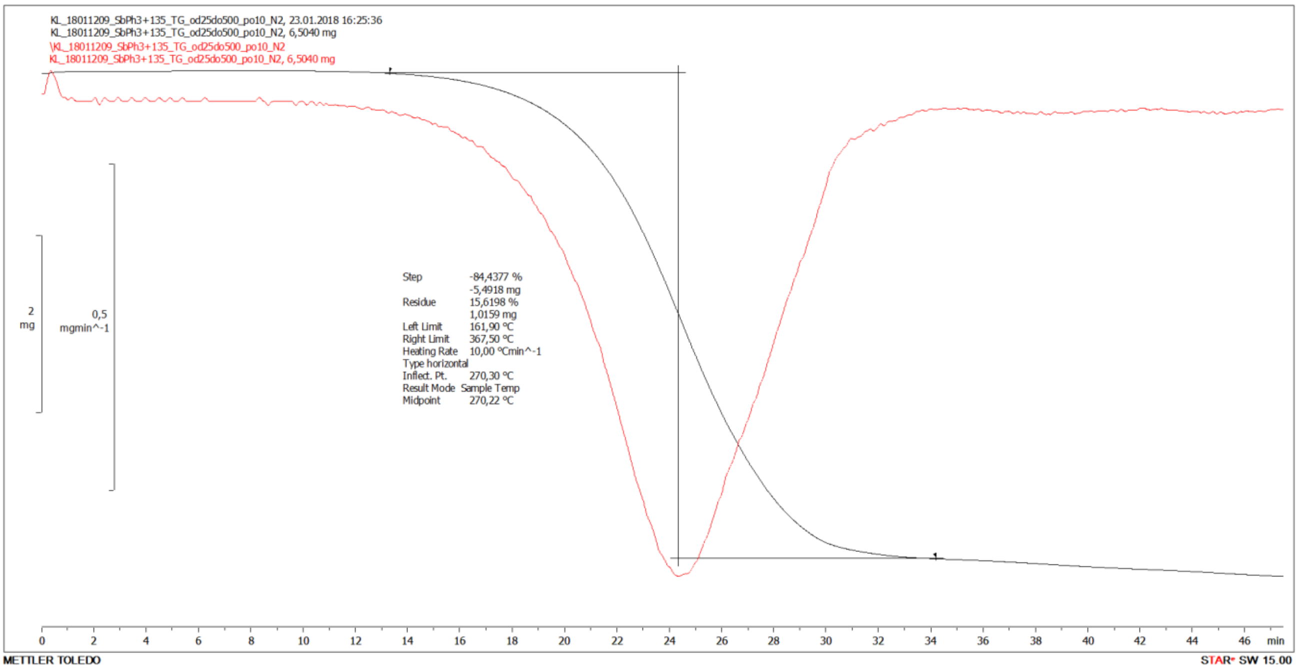

Supplementary Figure 26. TGA thermogram of (**tftib**)(**SbPh$_3$**) (25–500 °C, heating rate 10 °C min^{-1}).

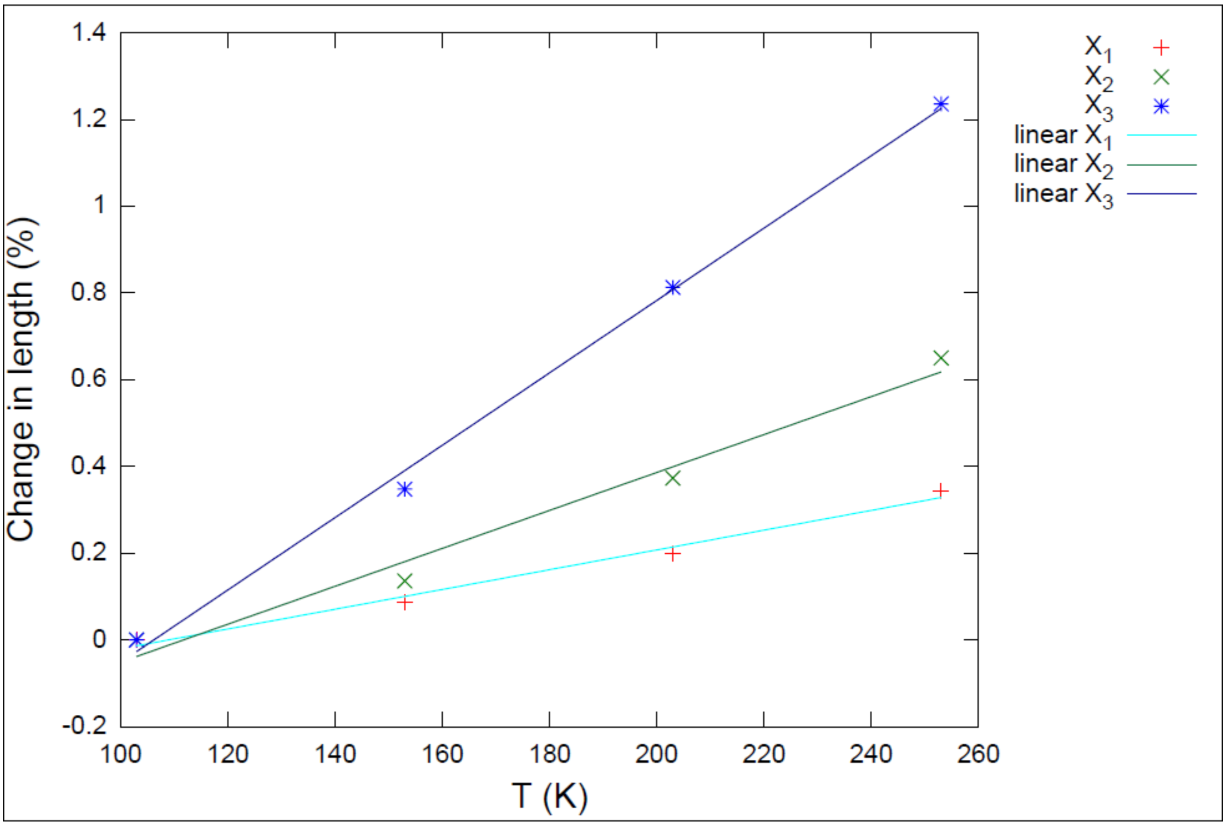

Supplementary Figure 27. Temperature dependence of the principal axes lengths for (**tftib**)(**PPh$_3$**).

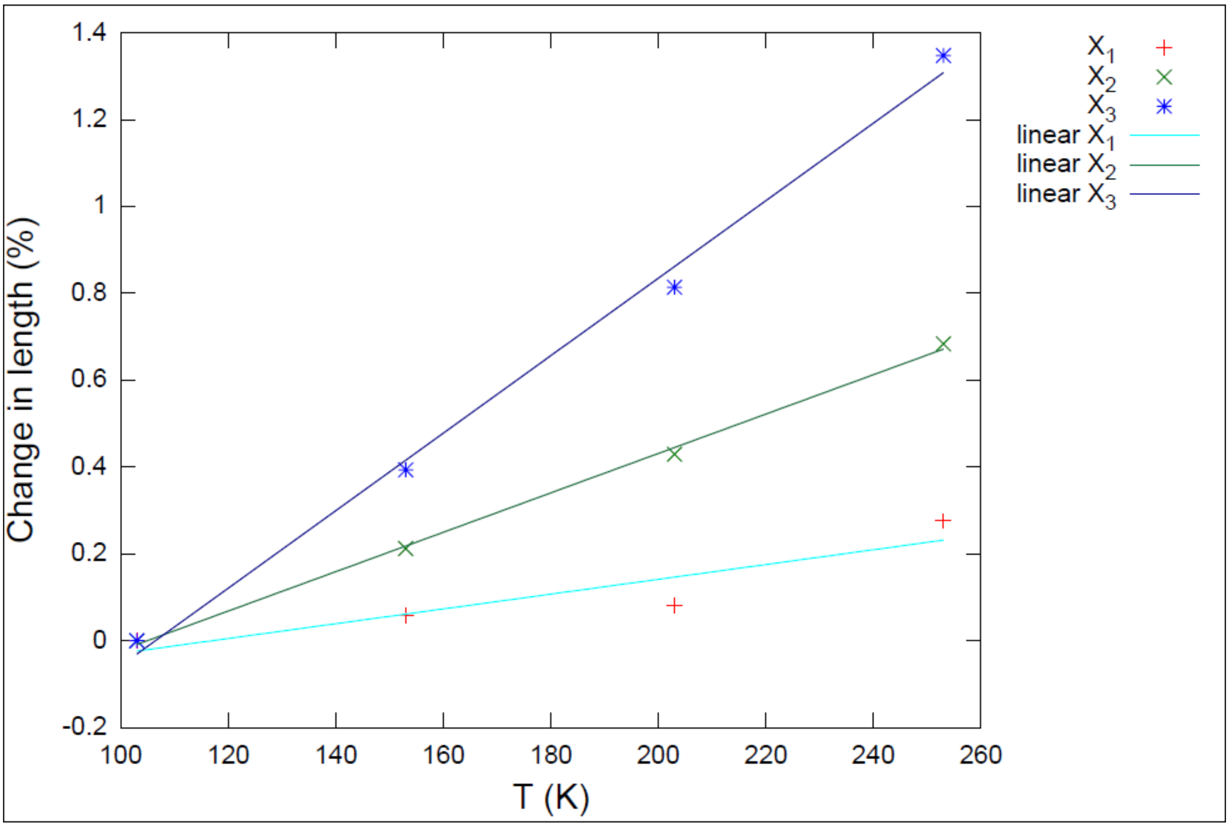

Supplementary Figure 28. Temperature dependence of the principal axes lengths for (**tftib**)(**$AsPh_3$**).

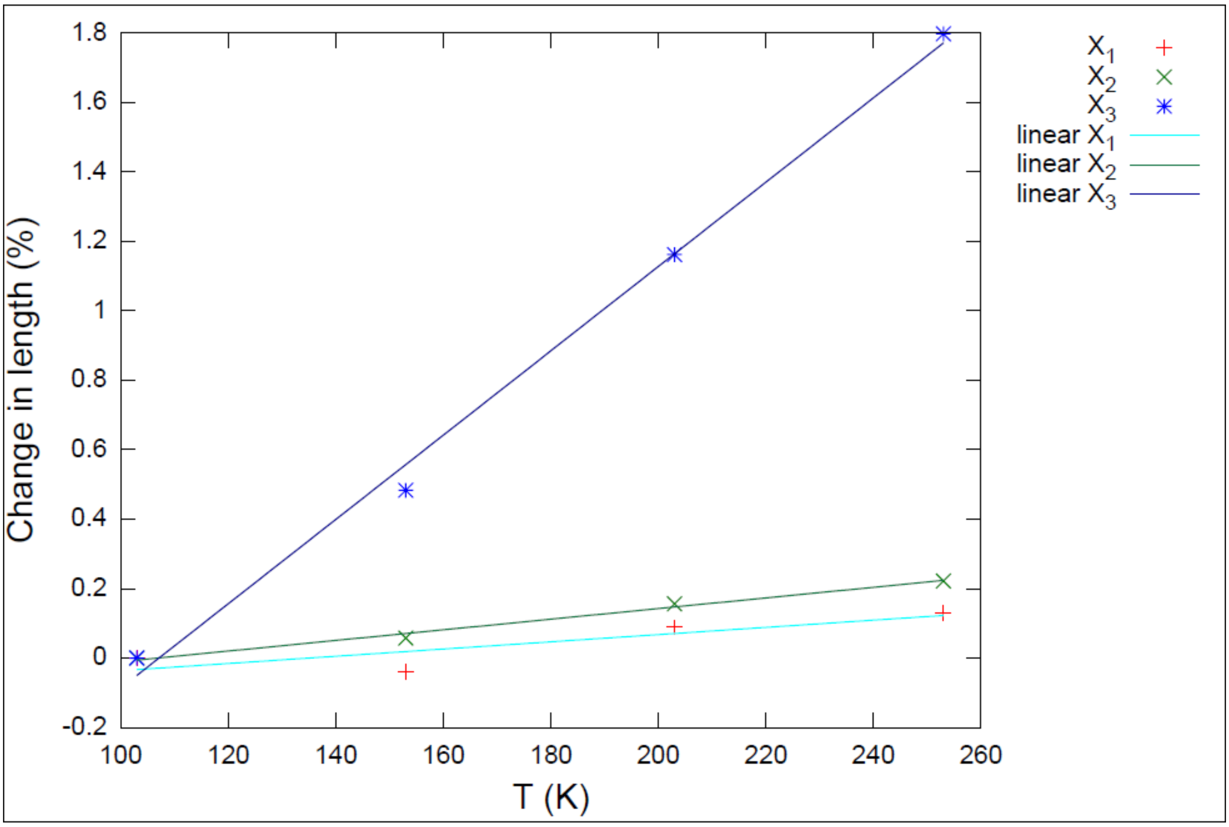

Supplementary Figure 29. Temperature dependence of the principal axes lengths for (**tftib**)(**$SbPh_3$**).

Supplementary Tables

Supplementary Table 1. Full crystallographic and general data for single crystals of (**tftib**)(**PPh_3**) at 103, 153, 203 and 253 K.

Cocrystal	(**tftib**)(**PPh_3**) @103 K	(**tftib**)(**PPh_3**) @153 K	(**tftib**)(**PPh_3**) @203 K	(**tftib**)(**PPh_3**) @253 K
CCDC Number	1850430	1850431	1850432	1850433
Formula	$C_{24}H_{15}F_3I_3P$	$C_{24}H_{15}F_3I_3P$	$C_{24}H_{15}F_3I_3P$	$C_{24}H_{15}F_3I_3P$
M_r	772.03	772.03	772.03	772.03
T (K)	103.0(1)	153.0(1)	203.0(1)	253.0(1)
Crystal system	triclinic	triclinic	triclinic	triclinic
Space group	$P\bar{1}$	$P\bar{1}$	$P\bar{1}$	$P\bar{1}$
a (Å)	9.1186(8)	9.1289(8)	9.1422(6)	9.1636(6)
b (Å)	10.8976(10)	10.9129(10)	10.9407(7)	10.9640(6)
c (Å)	13.3301(12)	13.3753(13)	13.4342(9)	13.4866(8)
α (°)	88.147(3)	88.096(3)	88.010(2)	87.9139(19)
β (°)	72.738(3)	72.693(3)	72.631(2)	72.560(2)
γ (°)	76.213(3)	76.213(3)	76.213(2)	76.292(2)
V (Å^3)	1227.46(19)	1234.5(2)	1244.53(14)	1255.01(13)
Z	2	2	2	2
ρ_{calc} (g cm^{-3})	2.089	2.077	2.060	2.043
μ (mm^{-1})	3.917	3.895	3.863	3.831
F(000)	720	720	720	720
Crystal size (mm^3)	0.498×0.410×0.371	0.498×0.410×0.371	0.498×0.410×0.371	0.557×0.436×0.406
θ range for data collection (°)	2.464–37.189	2.449–37.198	2.443–37.181	2.446–35.397
Reflections collected [R_{int}]	77694 [0.0439]	80338 [0.0403]	79044 [0.0366]	25349 [0.0303]
Reflections [$I > 2\sigma(I)$]	11995 [10770]	12049 [10057]	12160 [9531]	9957 [7342]
Data completeness (%)	99.7 to θ = 25.25°	99.8 to θ = 25.25°	99.6 to θ = 25.25°	98.9 to θ = 25.25°
Data/restraints/parameters	11995/0/281	12049/0/281	12160/0/281	9957/0/281
Goodness-of-fit on F^2	1.138	1.103	1.051	1.028
Final R for data with $I > 2\sigma(I)$	R_1 = 0.0266 wR_2 = 0.0498	R_1 = 0.0326 wR_2 = 0.0585	R_1 = 0.0375 wR_2 = 0.0714	R_1 = 0.0443 wR_2 = 0.0854
Final R for all data	R_1 = 0.0336 wR_2 = 0.0513	R_1 = 0.0464 wR_2 = 0.0618	R_1 = 0.0566 wR_2 = 0.0769	R_1 = 0.0675 wR_2 = 0.0939
Largest diff. peak/hole (e Å^{-3})	1.094/−1.100	1.514/−1.522	1.821/−1.688	1.998/−1.590
Extinction coefficient	0.0108(2)	0.0110(3)	0.0163(4)	0.0178(6)

Supplementary Table 2. Full crystallographic and general data for single crystals of (**tftib**)(**AsPh₃**) at 103, 153, 203 and 253 K.

Cocrystal	(**tftib**)(**AsPh$_3$**) @103 K	(**tftib**)(**AsPh$_3$**) @153 K	(**tftib**)(**AsPh$_3$**) @203 K	(**tftib**)(**AsPh$_3$**) @253 K
CCDC Number	1850434	1850435	1850436	1850437
Formula	$C_{24}H_{15}AsF_3I_3$	$C_{24}H_{15}AsF_3I_3$	$C_{24}H_{15}AsF_3I_3$	$C_{24}H_{15}AsF_3I_3$
M_r	815.98	815.98	815.98	815.98
T (K)	103.0(1)	153.0(1)	203.0(1)	253.0(1)
Crystal system	triclinic	triclinic	triclinic	triclinic
Space group	$P\bar{1}$	$P\bar{1}$	$P\bar{1}$	$P\bar{1}$
a (Å)	9.1477(8)	9.1584(7)	9.1673(7)	9.1849(18)
b (Å)	11.0051(9)	11.0276(9)	11.0467(8)	11.076(2)
c (Å)	13.3755(12)	13.4259(11)	13.4796(10)	13.548(3)
α (°)	88.515(3)	88.448(3)	88.367(3)	88.295(7)
β (°)	71.685(3)	71.598(3)	71.519(3)	71.520(7)
γ (°)	76.816(3)	76.842(3)	76.911(3)	76.922(7)
V (Å^3)	1243.10(19)	1251.36(18)	1259.64(16)	1272.0(4)
Z	2	2	2	2
ρ_{calc} (g cm^{-3})	2.180	2.166	2.151	2.130
μ (mm^{-1})	5.124	5.090	5.057	5.008
F(000)	756	756	756	756
Crystal size (mm^3)	0.556×0.455×0.374	0.556×0.455×0.374	0.556×0.455×0.374	0.556×0.455×0.374
θ range for data collection (°)	2.429–37.221	2.424–37.177	2.407–36.437	2.402–33.800
Reflections collected [R_{int}]	76455 [0.0600]	82754 [0.0598]	76812 [0.0564]	82425 [0.0524]
Reflections [$I > 2\sigma(I)$]	12152 [10890]	12252 [10227]	12069 [9100]	10181 [7487]
Data completeness (%)	99.7 to θ = 25.25°	99.7 to θ = 25.25°	99.8 to θ = 25.25°	99.7 to θ = 25.25°
Data/restraints/parameters	12152/0/281	12252/0/281	12069/0/281	10181/0/281
Goodness-of-fit on F^2	1.108	1.059	1.042	1.025
Final R for data with $I > 2\sigma(I)$	R_1 = 0.0313<br>wR_2 = 0.0667	R_1 = 0.0361<br>wR_2 = 0.0713	R_1 = 0.0428<br>wR_2 = 0.0823	R_1 = 0.0433<br>wR_2 = 0.0882
Final R for all data	R_1 = 0.0380<br>wR_2 = 0.0687	R_1 = 0.0492<br>wR_2 = 0.0751	R_1 = 0.0678<br>wR_2 = 0.0899	R_1 = 0.0667<br>wR_2 = 0.0972
Largest diff. peak/hole (e Å^{-3})	1.610/−1.744	1.610/−1.960	1.953/−1.795	2.100/−1.702
Extinction coefficient	0.0134(3)	0.0125(3)	0.0124(4)	0.0112(4)

Supplementary Table 3. Full crystallographic and general data for single crystals of (**tftib**)(**SbPh$_3$**) at 103, 153, 203 and 253 K.

Cocrystal	(**tftib**)(**SbPh$_3$**) @103 K	(**tftib**)(**SbPh$_3$**) @153 K	(**tftib**)(**SbPh$_3$**) @203 K	(**tftib**)(**SbPh$_3$**) @253 K
CCDC Number	1850438	1850439	1850440	1850441
Formula	$C_{24}H_{15}F_3I_3Sb$	$C_{24}H_{15}F_3I_3Sb$	$C_{24}H_{15}F_3I_3Sb$	$C_{24}H_{15}F_3I_3Sb$
M_r	862.81	862.81	862.81	862.81
T (K)	103.0(1)	153.0(1)	203.0(1)	253.0(1)
Crystal system	triclinic	triclinic	triclinic	triclinic
Space group	$P\bar{1}$	$P\bar{1}$	$P\bar{1}$	$P\bar{1}$
a (Å)	9.1863(10)	9.1869(11)	9.2011(7)	9.2067(6)
b (Å)	11.2726(12)	11.2785(14)	11.2853(8)	11.2930(8)
c (Å)	13.3561(14)	13.4077(15)	13.4978(9)	13.5684(8)
α (°)	89.190(4)	89.071(4)	89.047(2)	88.927(2)
β (°)	70.066(4)	70.160(4)	70.167(2)	70.283(2)
γ (°)	78.455(4)	78.406(4)	78.429(2)	78.422(2)
V (Å^3)	1271.7(2)	1278.1(3)	1289.63(16)	1299.16(15)
Z	2	2	2	2
ρ_{calc} (g cm^{-3})	2.253	2.242	2.222	2.206
μ (mm^{-1})	4.757	4.734	4.691	4.657
F(000)	792	792	792	792
Crystal size (mm^3)	0.527×0.365×0.350	0.527×0.365×0.350	0.527×0.365×0.350	0.527×0.365×0.350
θ range for data collection (°)	2.387–37.141	2.385–37.216	2.378–35.696	2.374–34.670
Reflections collected [R_{int}]	70256 [0.0448]	83522 [0.0496]	70987 [0.0416]	79444 [0.0421]
Reflections [$I > 2\sigma(I)$]	12342 [10920]	12511 [10199]	11851 [9363]	11102 [8104]
Data completeness (%)	99.7 to θ = 25.25°	99.8 to θ = 25.25°	99.4 to θ = 25.25°	99.6 to θ = 25.25°
Data/restraints/parameters	12342/0/281	12511/0/281	11851/0/281	11102/0/281
Goodness-of-fit on F^2	1.146	1.095	1.047	1.042
Final R for data with $I > 2\sigma(I)$	R_1 = 0.0312<br>wR_2 = 0.0589	R_1 = 0.0373<br>wR_2 = 0.0668	R_1 = 0.0404<br>wR_2 = 0.0819	R_1 = 0.0462<br>wR_2 = 0.0819
Final R for all data	R_1 = 0.0397<br>wR_2 = 0.0608	R_1 = 0.0545<br>wR_2 = 0.0709	R_1 = 0.0575<br>wR_2 = 0.0876	R_1 = 0.0575<br>wR_2 = 0.0876
Largest diff. peak/hole (e Å^{-3})	1.480/−1.725	2.178/−2.266	2.652/−2.410	2.785/−2.365
Extinction coefficient	0.0077(2)	0.0072(2)	0.0106(4)	0.0094(4)

Supplementary Table 4. Electronic energies of crystalline materials and cocrystal formation energies.

Cocrystal	Electronic energy corrected for BSSE / Ha			Cocrystal energy of formation / kJ mol^{-1}
	**EPh$_3$**	**tftib**	(**tftib**)(**EPh$_3$**)	
(**tftib**)(**PPh$_3$**)	−1036.1484	−1415.2574	−2451.4116	−15.40
(**tftib**)(**AsPh$_3$**)	−2930.7083	−1415.2574	−4345.9701	−11.38
(**tftib**)(**SbPh$_3$**)	−935.0740	−1415.2574	−2350.3343	−7.36
(**tftib**)(**BiPh$_3$**)	−909.4045	−1415.2574	−2324.6615	+1.27

Supplementary Table 5. Electronic energies of halogen-bonded dimers, geometry taken from optimized cocrystal structures.

Dimer	Electronic energy corrected for BSSE / Ha			Dimer energy of formation / kJ mol^{-1}
	**EPh$_3$**	**tftib**	(**tftib**)(**EPh$_3$**)	
(**tftib**)(**PPh$_3$**)	−1036.1535	−1415.2491	−2451.4091	−16.96
(**tftib**)(**AsPh$_3$**)	−2930.7092	−1415.2496	−4345.9638	−13.12
(**tftib**)(**SbPh$_3$**)	−935.0729	−1415.2500	−2350.3277	−12.88
(**tftib**)(**BiPh$_3$**)	−909.4034	−1415.2498	−2324.6553	−5.65

Supplementary Table 6. Electronic energies of (**tftib**)(**$BiPh_3$**) halogen-bonded dimer, corrected for spin-orbit coupling. Dimer geometry was taken from the optimized putative cocrystal structure.

Calculation	Binding energy, relative to isolated atoms / Ha			Dimer energy of formation / kJ mol^{-1}
	**$BiPh_3$**	**tftib**	(**tftib**)(Bi**Ph_3**)	
non-relativistic DFT	−12.4761	−4.2636	−16.7421	−6.11
spin-orbit ZORA	−57.3936	−11.2274	−68.6234	−6.21

Supplementary Table 7. Electronic energies of halogen-bonded dimers in gas-phase geometry.

Dimer	Electronic energy corrected for BSSE / Ha			Dimer energy of formation / kJ mol^{-1}
	**EPh_3**	**tftib**	(**tftib**)(**EPh_3**)	
(**tftib**)(**NPh_3**)	−749.5569	−1415.2517	−2164.8139	−13.87
(**tftib**)(**PPh_3**)	−1036.1547	−1415.2501	−2451.4136	−22.79
(**tftib**)(**$AsPh_3$**)	−2930.7118	−1415.2501	−4345.9679	−15.66
(**tftib**)(**$SbPh_3$**)	−935.0753	−1415.2504	−2350.3316	−15.67
(**tftib**)(**$BiPh_3$**)	−909.4061	−1415.2502	−2324.6590	−7.23

Supplementary Table 8. Comparison of halogen bond geometric parameters in the experimental (measured at 103 K) and DFT-optimized cocrystal structures.

Cocrystal	$d_{XB,calc}$ / Å		$\angle_{C\text{-}I\cdots E}$ / °	
	Experimental (103 K)	Calculated	Experimental (103 K)	Calculated
(**tftib**)(**PPh_3**)	3.313	3.363	165.33	160.71
(**tftib**)(**$AsPh_3$**)	3.421	3.529	166.02	159.99
(**tftib**)(**$SbPh_3$**)	3.575	3.577	168.28	162.74

Supplementary Table 9. Comparison of the experimental and DFT-optimized unit cell parameters (measured at 103 K) of halogen-bonded cocrystals.

	(**tftib**)(**PPh_3**)			(**tftib**)(**$AsPh_3$**)			(**tftib**)(**$SbPh_3$**)		
Unit cell parame-ters	Experimental (103 K)	Calculated	Relative error	Experimental (103 K)	Calculated	Relative Error	Experimental (103 K)	Calculated	Relative error
a (Å)	9.1186(8)	8.9352	−2.0%	9.1477(8)	8.9825	−1.8%	9.1863(10)	9.0227	−1.8%
b (Å)	10.8976(10)	10.4704	−3.9%	11.0051(9)	10.5712	−3.9%	11.2726(12)	10.7773	−4.4%
c (Å)	13.3301(12)	12.8346	−3.7%	13.3755(12)	12.9834	−2.9%	13.3561(14)	12.9852	−2.8%
α (°)	88.147(3)	90.681	+2.9%	88.515(3)	91.107	+2.9%	89.190(4)	91.661	+2.8%
β (°)	72.738(3)	75.903	+4.3%	71.685(3)	74.896	+4.5%	70.066(4)	74.815	+6.8%
γ (°)	76.213(3)	75.733	−0.6%	76.816(3)	75.841	−1.3%	78.455(4)	76.670	−2.3%
V ($Å^3$)	1227.46(19)	1125.36	−8.3%	1243.10(19)	1149.54	−7.5%	1271.7(2)	1180.37	−7.2%

Supplementary Notes

Supplementary Note 1:

Supplementary Datasets 1-9 contain the coordinates for DFT optimized crystal structures of the starting materials, observed cocrystals (**tftib**)(**PPh_3**), (**tftib**)(**$AsPh_3$**), (**tftib**)(**$SbPh_3$**) and the putative cocrystal (**tftib**)(**$BiPh_3$**) as separate CIF files:

Dataset 1: DFT_optimized_crystal_structure_Reactant_tftib.cif

Dataset 2: DFT_optimized_crystal_structure_Reactant_PPh3.cif

Dataset 3: DFT_optimized_crystal_structure_Reactant_AsPh3.cif

Dataset 4: DFT_optimized_crystal_structure_Reactant_SbPh3.cif

Dataset 5: DFT_optimized_crystal_structure_Reactant_BiPh3.cif

Dataset 6: DFT_optimized_cocrystal_structure_(tftib)(PPh3).cif

Dataset 7: DFT_optimized_cocrystal_structure_(tftib)(AsPh3).cif

Dataset 8: DFT_optimized_cocrystal_structure_(tftib)(SbPh3).cif

Dataset 9: DFT_optimized_cocrystal_structure_(tftib)(BiPh3).cif

Supplementary Note 2:

Supplementary Datasets 10-14 contain the geometries of optimized halogen-bonded dimers in the gas phase as separate XYZ files:

Dataset 10: gas-phase_(tftib)(NPh3)_optimized_dimer.xyz

Dataset 11: gas-phase_(tftib)(PPh3)_optimized_dimer.xyz

Dataset 12: gas-phase_(tftib)(AsPh3)_optimized_dimer.xyz

Dataset 13: gas-phase_(tftib)(SbPh3)_optimized_dimer.xyz

Dataset 14: gas-phase_(tftib)(BiPh3)_optimized_dimer.xyz
