# Supplementary material for: Halogen-bonded cocrystallization with phosphorus, arsenic and antimony acceptors
Source: Nat Commun. 2019 Jan 4;10:61. doi: 10.1038/s41467-018-07957-6 (PMC6320372; doi:10.1038/s41467-018-07957-6)
Supplement: Supplementary file 17 — Description of Additional Supplementary Files [file 41467_2018_7957_MOESM17_ESM.docx]

Description of Additional Supplementary Files

**Supplementary Data 1:** DFT optimized crystal structure for reactant tftib in CIF format.

**Supplementary Data 2:** DFT optimized crystal structure for reactant PPh3 in CIF format.

**Supplementary Data 3:** DFT optimized crystal structure for reactant AsPh3 in CIF format.

**Supplementary Data 4:** DFT optimized crystal structure for reactant SbPh3 in CIF format.

**Supplementary Data 5:** DFT optimized crystal structure for reactant BiPh3 in CIF format.

**Supplementary Data 6:** DFT optimized crystal structure for the cocrystal (tftib)(PPh3) in CIF format.

**Supplementary Data 7:** DFT optimized crystal structure for the cocrystal (tftib)(AsPh3) in CIF format.

**Supplementary Data 8:** DFT optimized crystal structure for the cocrystal (tftib)(SbPh3) in CIF format

**Supplementary Data 9:** DFT optimized crystal structure for the cocrystal (tftib)(BiPh3) in CIF format.

**Supplementary Data 10:** Geometry of the gas-phase optimized dimer (tftib)(NPh3) in XYZ format.

**Supplementary Data 11:** Geometry of the gas-phase optimized dimer (tftib)(PPh3) in XYZ format.

**Supplementary Data 12:** Geometry of the gas-phase optimized dimer (tftib)(AsPh3) in XYZ format.

**Supplementary Data 13:** Geometry of the gas-phase optimized dimer (tftib)(SbPh3) in XYZ format

**Supplementary Data 14:** Geometry of the gas-phase optimized dimer (tftib)(BiPh3) in XYZ format
